# Methods of analysis of chloroplast genomes of C_3_, Kranz type C_4_ and Single Cell C_4_ photosynthetic members of Chenopodiaceae

**DOI:** 10.1186/s13007-020-00662-w

**Published:** 2020-08-31

**Authors:** Richard M. Sharpe, Bruce Williamson-Benavides, Gerald E. Edwards, Amit Dhingra

**Affiliations:** 1 Department of Horticulture, Washington State University, Pullman, WA 99164 USA; 2 Molecular Plants Sciences, Washington State University, Pullman, WA 99164 USA; 3 School of Biological Sciences, Washington State University, Pullman, WA 99164 USA

## Abstract

**Background:**

Chloroplast genome information is critical to understanding forms of photosynthesis in the plant kingdom. During the evolutionary process, plants have developed different photosynthetic strategies that are accompanied by complementary biochemical and anatomical features. Members of family Chenopodiaceae have species with C_3_ photosynthesis, and variations of C_4_ photosynthesis in which photorespiration is reduced by concentrating CO_2_ around Rubisco through dual coordinated functioning of dimorphic chloroplasts. Among dicots, the family has the largest number of C_4_ species, and greatest structural and biochemical diversity in forms of C_4_ including the canonical dual-cell Kranz anatomy, and the recently identified single cell C_4_ with the presence of dimorphic chloroplasts separated by a vacuole. This is the first comparative analysis of chloroplast genomes in species representative of photosynthetic types in the family.

**Results:**

Methodology with high throughput sequencing complemented with Sanger sequencing of selected loci provided high quality and complete chloroplast genomes of seven species in the family and one species in the closely related Amaranthaceae family, representing C_3_, Kranz type C_4_ and single cell C_4_ (SSC_4_) photosynthesis six of the eight chloroplast genomes are new, while two are improved versions of previously published genomes. The depth of coverage obtained using high-throughput sequencing complemented with targeted resequencing of certain loci enabled superior resolution of the border junctions, directionality and repeat region sequences. Comparison of the chloroplast genomes with previously sequenced plastid genomes revealed similar genome organization, gene order and content with a few revisions. High-quality complete chloroplast genome sequences resulted in correcting the orientation the LSC region of the published *Bienertia sinuspersici* chloroplast genome, identification of stop codons in the rpl23 gene in *B. sinuspersici* and *B. cycloptera*, and identifying an instance of IR expansion in the *Haloxylon ammodendron* inverted repeat sequence. The rare observation of a mitochondria-to-chloroplast inter-organellar gene transfer event was identified in family Chenopodiaceae.

**Conclusions:**

This study reports complete chloroplast genomes from seven Chenopodiaceae and one Amaranthaceae species. The depth of coverage obtained using high-throughput sequencing complemented with targeted resequencing of certain loci enabled superior resolution of the border junctions, directionality, and repeat region sequences. Therefore, the use of high throughput and Sanger sequencing, in a hybrid method, reaffirms to be rapid, efficient, and reliable for chloroplast genome sequencing.

## Introduction

Plastids convert light energy into chemical energy and are an essential site for the biosynthesis of pigments, lipids, several amino acids and vitamins [1, 2]. Comparative genomics studies have facilitated the understanding of chloroplast genome organization and phylogenetic relationships [3–5]. Additionally, availability of chloroplast genome sequences can be useful for constructing transformation vectors to enable chloroplast transformation via homologous recombination [6, 7].

Higher plant chloroplast genomes possess a characteristic organization comprising a Large Single Copy (LSC), a Small Single Copy (SSC) and two Inverted Repeat (IRa and IRb) regions, with only a few exceptions, e.g. in *Pisum sativum* and some other legumes [8–10]. Several methods have been used to sequence chloroplast genomes in plants, including primer walking [11–14] and high-throughput sequencing (HTS) [15]. HTS, both with isolated chloroplast DNA [16–18] and total cellular DNA [19–21], has been employed to generate physical maps of the chloroplast genome. However, the junctions of LSC/IRa, IRa/SSC, SSC/IRb and IRb/LSC need to be resolved using additional experimentation [22]. Genome sequencing and subsequent assembly of the chloroplast genome can be challenging due to variable IR borders; presence of chloroplast genome sequences in the nuclear genome; sequence homology between chloroplast and mitochondrial genes, such as the NAD(P)H and NADH dehydrogenase genes; as well as the NAD(P)H genes being distributed throughout the chloroplast genome [3, 23–28].

Chloroplasts, the green plastids in plants, are the site of photosynthesis where Ribulose-1,5-bisphosphate carboxylase/oxygenase (Rubisco), captures CO_2_ with synthesis of 3-phosphoglyceric acid (3PGA) in the Calvin-Benson cycle, leading to the synthesis of carbohydrates and cellular constituents. Three major types of oxygenic photosynthesis are known to date: C_3_, C_4_, and Crassulacean acid metabolism (CAM). In C_3_ plants, Rubisco directly fixes atmospheric CO_2_ introducing carbon into the Calvin-Benson cycle. In C_4_ and CAM photosynthesis, CO_2_ is first captured by phosphoenolpyruvate carboxylase (PEPC) with synthesis of 4-carbon organic acids which are sequestered in a spatial manner in C_4_ plants and a temporal manner in CAM plants. Decarboxylation of the 4-carbon organic acid generates a CO_2_-rich environment around Rubisco [29]. This mechanism suppresses the oxygenation reaction by Rubisco and the subsequent energetically-wasteful photorespiratory pathway. C_4_ plants function with spatial separation of two types of chloroplasts, one type supports the fixation of atmospheric CO_2_ by PEPC and synthesis of C_4_ acids, while the other type utilizes the CO_2_ generated from decarboxylation of C_4_ acids in the Calvin Benson cycle. In Kranz type C_4_ plants mesophyll chloroplasts support fixation of atmospheric CO_2_ by PEPC, while bundle sheath chloroplasts utilize CO_2_ generated by decarboxylation of C_4_ acids. The unique single-cell C_4_ (SCC_4_) plants perform C_4_ photosynthesis within individual chlorenchyma cells with spatial separation of two types of chloroplasts. One type supports capture of atmospheric CO_2_ by PEPC and the other assimilates the CO_2_ generated by decarboxylation of C_4_ acids in the Benson-Calvin cycle [30–32].

Among dicot families, the Chenopodiaceae and Amaranthaceae families have by far the largest number (~ 800) of C_4_ species, with up to 15 distinct lineages [33]. Although they are currently recognized as separate families in a clade, they are known to be closely related [34]. Chenopodiaceae species are acclimated to diverse ecosystems from xeric to more temperate salt marshes, including highly saline soils; while Amaranthus species predominantly occur in tropical and subtropical regions. The Chenopodiaceae family is very diverse, with six structural forms of Kranz anatomy present among its members [35]. Furthermore, it is the only family known to have SCC_4_ species [34]. Phylogenetic analyses have identified independent origins of C_4_ photosynthesis. In particular, the results allude to the unique independent origins of C_4_ in subfamily Suaedoideae, including Kranz C_4_ anatomy in *Suaeda* species and two independent origins of the SCC_4_ system in *Bienertia* and *Suaeda* [33, 36–39]. In general the causation of these independent events is hypothesized to be a result of the harsh environments induced by global climate change and periodic reductions in CO_2_ content over the past 35 million years [40, 41].

In this study, complete chloroplast genome sequences for seven Chenopodiaceae species and one Amaranthaceae species were generated using whole leaf tissue genomic DNA (gDNA) via HTS complemented with Sanger sequencing of targeted loci. The species analyzed were: *Bassia muricata* (C_4_-Kochioid anatomy, tribe Camphorosmoideae), *Haloxylon ammodendron* (C_4_-Salsoloid anatomy, tribe Salsoleae), *Bienertia cycloptera* (C_4_: SCC_4_-tribe Suaedeae), *Bienertia sinuspersici* (C_4_: SCC_4_-tribe Suaedeae)*, Suaeda aralocaspica* (SCC_4_-tribe Suaedeae), *Suaeda eltonica* (C_4_-Schoberioid type anatomy, tribe Suaedeae), and* Suaeda maritima* (C_3_-tribe Suaedeae)*.* The chloroplast genome from *Amaranthus retroflexus* (C_4_-Atriplicoid type anatomy, family Amaranthaceae, tribe Amarantheae), was also sequenced and used for comparative analysis. These dicot species include representative species having C_3_-type photosynthesis with monomorphic chloroplasts, and C_4_ species having dimorphic chloroplasts for C_4_ function including its development in Kranz anatomy versus individual chlorenchyma cells. The purpose of the present study was to determine among these representative dicot species whether the chloroplast genomes between C_3_ and C_4_ species, and the chloroplast genomes between the various forms of C_4_, are highly conserved (in size and composition), and the degree of difference between the species.

## Results and discussion

### Genome sequencing and assembly

A summary of the sequencing data obtained from Illumina sequencing and assembly of *A. retroflexus, B. muricata, B. cycloptera*, *B. sinuspersici, H. ammodendron, S. aralocaspica, S. eltonica,* and *S. maritima* chloroplast genomes is presented in Table 1. Three large contigs with overlapping 5′ and 3′ regions were generated during genome assembly for *A. retroflexus, B. muricata, B. cycloptera, B. sinuspersici, H. ammodendron, S. aralocaspica,* and *S. maritima.* These three contigs were identified as LSC, SSC, and IR via BLAST homology alignment [42], GE-Seq—Annotation of Organellar Genomes [43] and DOGMA gene identity prediction [44]. The overlapping regions were present at all four possible junctions when the IR region was reverse complemented (LSC-IR, IR-SSC, SSC-IR, and IR-LSC). These overlapping areas ranged from 19 to 51 nt (illustrated in Additional file 1: Figure S1 with *B. cycloptera*). The directionality of the LSC, SSC and IR, and all overlapping aligned junctions were validated via Sanger sequencing of both strands of the amplicons generated from these regions (Additional file 2: Table S1; Additional file 1: Figure S1). For *S. eltonica*, the LSC-IRa and IRb-LSC overlapping regions were 23 nt long and were validated with Sanger sequencing (Additional file 2: Table S1). The IRa-SSC and SSC-IRb sections were both missing a 1,475 nt section in the IRa and IRb borders. The 300 nt sequence contiguous to the 1475 nt section had a low GC content of 19%. A possible cause of the shortened contig flanking the IR-1475 area may be due to the low GC content value which could impact the accuracy of the HTS genome assembly [45]. The 1,475 nt section was sequenced by primer walking and Sanger sequencing (Additional file 2: Table S1). The GC content in the 1,475 nt region and IR was 31.3 and 42.1%, respectively.

**Table 1:** Sequencing and assembly data when length fraction and similarity fraction parameters were set to 80 and 90 respectively during read mapping in the chloroplast genomes of eight Chenopod species

Variable	Species							
	*A. retroflexus*	*B. cycloptera*	*B. muricata*	*B. sinuspersici*	*H. ammodendron*	*S. aralocaspica*	*S. eltonica*	*S. maritima*
Total number of reads	94,491,120	73,061,587	61,098,096	80,215,373	81,126,072	86,825,544	66,358,800	87,411,736
Mean read length (nt)	78.98	79.04	79.44	79.66	78.5	78.83	78.35	79.88
Minimum coverage (bases)	22	12	37	39	12	55	25	33
Maximum coverage (bases)	29,721	6,135	23,533	15,558	15,284	23,266	14,652	6,991
Average coverage (bases)	3,649.67	1,553.12	3,204.28	5,998.08	1,357.20	4,864.44	1,591.56	4,111.85
Total (%) of reads assemble to genome	7.37	4.12	10.01	14.39	3.44	10.42	4.55	8.95

The average base depth of coverage for the eight assembled chloroplast genomes ranged from 1553- to 5998-fold. For accurate assembly a minimum of 30–40 × sequence coverage is recommended [46–48]. In this study, the only areas with less than 40 × average coverage were identified in the last 1–3 nucleotides of the IRb sequence for each of the eight genomes. This is expected due to the assembler algorithm parameters. The end of the IRb and the beginning of the LSC were concatenated and these sections were remapped. Remapped coverage results were reported to be above 40 × for the IRb ends and surrounding areas. The eight assembled genomes (0.8/0.9 for the read length fraction/similarity fraction mapping) were also compared with a more stringent remapping of the reads to the contigs of 0.99/0.99 length fraction/similarity fraction. Analyses with both levels of stringency show almost identical assembly minimum-coverage and average-coverage for the eight species sequenced in this study (Additional file 3: Figure S2).

Overall, the assembly and subsequent Sanger sequencing-based validation generated high quality and complete chloroplast genomes with all possessing a quadripartite structure as reported in other land plant species.

### Size, organization and gene content of the chloroplast genomes

**Fig. 1:**
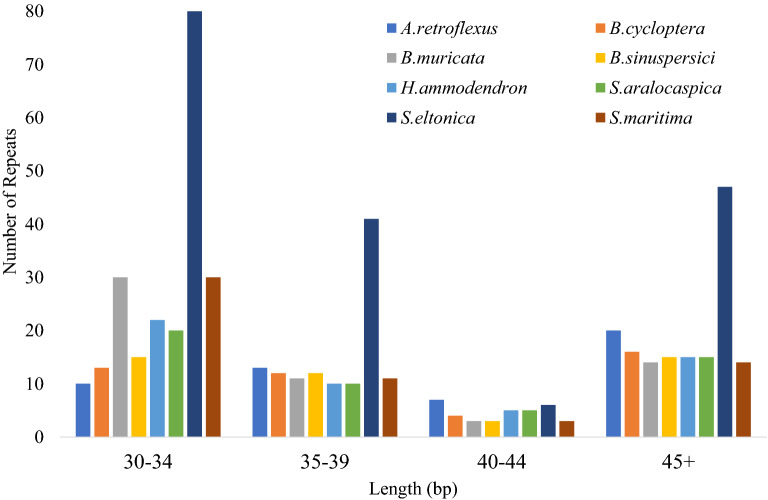
Histogram of number of repeated sequences (> /30 nt) in length identified with REPuter for nine chloroplast genomes.

The size of the chloroplast genomes from the eight species ranged from 146,634 to 161,251 nt (Table 2). As expected, each chloroplast genome included a pair of inverted repeat regions, IRa and IRb, separated by an SSC and an LSC region (Table 2 and Additional file 4: Figure S3). With one exception, the size of the IRs ranged from 23,461 to 25,213 nt. (Table 2). The *H. ammodendron* inverted repeat sequence presented an instance of IR length expansion (29,061 nt) compared to the other seven species. The GC content was similar among the eight species and for all the plastomes, LSC, SSC and IRs it ranged from 36.4–36.6, 34.1–34.6, 29.1–30.2 and 42.1–43.0%, respectively (Table 2). All chloroplast genomes contained a similar number of protein coding, ribosomal, and tRNA genes. The number of genes and tRNAs ranged from 113 to 116 and 27 to 29, respectively in the eight genomes (Table 3 and Additional file 4: Figure S3). For seven of eight species, 60.1–61.9% of the chloroplast sequence consisted of coding region, which included 52.7–54.3% of protein coding genes and 7.4–7.9% of RNA genes. The *S. eltonica* chloroplast genome was composed of 56.8% coding region including 48.9% of protein coding genes and 7.9% of RNA genes. This difference between *S. eltonica* and the rest of chloroplast genomes is possibly due to the higher repeat content in intergenic sequences of the *S. eltonica* chloroplast genome (Table 4 and Fig. 1).

**Table 2:** A summary of the complete chloroplast genome, IR, LSC and SSC length (nt) and GC content from *A. retroflexus, B. muricata, B. cycloptera, B. sinuspersici, H. ammodendron, S. aralocaspica, S. eltonica*, and *S. maritima*

Species	Complete chloroplast genome		IR		LSC		SSC	
	Size (bp)	GC content (%)	IRs size (bp)	GC content (%)	LSC size (bp)	GC content (%)	SSC size (bp)	GC content (%)
*A. retroflexus*	150,786	36.67	24,353	42.64	83,963	34.51	18,117	30.20
*B. muricata*	151,593	36.61	24,355	43.00	84,288	34.50	18,595	29.42
*B. cycloptera*	153,341	36.50	24,942	42.92	84,541	34.42	18,916	29.69
*B. sinuspersici*	153,334	36.65	24,949	42.97	84,490	34.57	18,946	29.49
*H. ammodendron*	161,251	36.42	29,061	42.90	84,236	34.18	18,893	29.42
*S. aralocaspica*	146,634	36.53	23,461	42.94	81,878	34.42	17,834	29.30
*S. eltonica*	148,729	36.44	24,585	42.11	80,218	34.69	19,341	29.20
*S. maritima*	152,011	36.45	25,213	42.72	83,482	34.11	18,103	29.17

**Table 3:** A summary of the number of genes in the eight Chenopodiaceae chloroplast genomes

Species name	CDS genes	rRNA	tRNA	genes w Introns	tRNA w Introns	Total genes
*A. retroflexus*	83	4	29	rps12, rps16, atpF, rpoC1, ycf3, clpP, ndhB, ndhA, ndhB	trnK-UUU, trnS-AGA, trnS-CGA, trnL-UAA, trnV-UAC, trnR-UCU, trnA-UGC, trnE-UUC, trnW-CCA, trnStop-UUA, trnC-ACA, trnD-GUC	116
*B. cycloptera*	83	4	27	clpP, rps12, ycf3, rpoC1, atpF, rps16, ndhB, ndhA, ndhB	trnT-UGU, trnC-ACA, trnL-UAA, trnF-GAA, trnS-CGA, trnK-UUU, trnE-UUC, trnA-UGC, trnW-CCA, trnW-CCA, trnA-UGC, trnE-UUC	114
*B. muricata*	84	4	28	rps12, rps16, atpF, rpoC1, ycf3, rps12, clpP, ndhB, ndhA	trnK-UUU, trnS-AGA, trnS-CGA, trnL-UAA, trnV-UAC, trnE-UUC, trnA-UGC, trnR-UCU, trnW-CCA, trnA-UGC, trnE-UUC	116
*B. sinuspersici*	83	4	27	clpP, rps12, ycf3, rpoC1, atpF, rps16, ndhB, ndhA, ndhB	trnT-UGU, trnC-ACA, trnL-UAA, trnF-GAA, trnS-CGA, trnK-UUU, trnE-UUC, trnA-UGC, trnW-CCA, trnW-CCA, trnA-UGC, trnE-UUC	114
*H. ammodendron*	82	4	27	clpP, rps12, ycf3, rpoC1, atpF, rps16, ndhB, ndhA, ndhB	trnC-ACA, trnL-UAA, trnS-CGA, trnK-UUU, trnE-UUC, trnA-UGC, trnW-CCA, trnW-CCA, trnA-UGC, trnE-UUC	113
*S. aralocaspica*	84	4	27	clpP, rps12, ycf3, rpoC1, atpF, rps16, ndhB, ndhA, ndhB	trnV-UAC, trnL-UAA, trnG-CCC, trnK-UUU, trnK-UUU, trnE-UUC, trnA-UGC, trnW-CCA, trnW-CCA, trnA-UGC, trnE-UUC	115
*S. eltonica*	83	4	28	clpP, rps12, ycf3, rpoC1, atpF, rps16, ndhB, ndhA, ndhB	trnA-GGC, trnV-UAC, trnL-UAA, trnS-CGA, trnK-UUU, trnE-UUC, trnI-GAU, trnA-UGC, trnA-UGC, trnE-UUC	115
*S. maritima*	84	4	28	clpP, rps12, ycf3, rpoC1, atpF, rps16, ndhB, ndhA, ndhB	trnC-ACA, trnL-UAA, trnK-CUU, trnS-CGA, trnK-UUU, trnE-UUC, trnA-UGC, trnW-CCA, trnW-CCA, trnA-UGC, trnE-UUC	116

**Table 4:** Distribution of repeated sequences (> /30 nt) among intergenic regions, exons and introns in eight chloroplast genomes

Location	*A. retroflexus*		*B. cycloptera*		*B. muricata*		*B. sinuspersici*		*H. ammodendron*		*S. aralocaspica*		*S. eltonica*		*S. maritima*	
	Total	%	Total	%	Total	%	Total	%	Total	%	Total	%	Total	%	Total	%
Intergenic	17	50.00	12	38.71	16	33.33	13	40.63	25	54.35	13	34.21	136	78.16	20	41.67
Exons	14	41.18	16	51.61	32	66.67	16	50.00	21	45.65	22	57.89	35	20.11	25	52.08
Introns	3	8.82	3	9.68	0	0.00	3	9.38	0	0.00	3	7.89	3	1.72	3	6.25

Gene order and content were largely conserved among the eight chloroplast genomes in this study. However, some structural rearrangements, gene losses and IR expansions were identified. The genes ycf15, ycf68, and rpl23 were identified as pseudogenes due to the presence of internal stop codons. The ycf15 and ycf68 genes are quite commonly classified as pseudogenes in angiosperms [23, 49]. The rpl23 is also classified as a pseudogene in some species such as the *Fagopyrum* spp., buckwheat, and spinach as well as *Suaeda* and *Haloxylon* species [22, 23, 50, 51]. In *S. eltonica,* rpl23 was not predicted to be in the chloroplast genome by GeSeq but it was identified as a pseudogene via the BLAST sequence analysis [42]. No stop codons were identified in the rpl23 of a previously published *B. sinuspersici* chloroplast genome [52]. In this study, 4 stop codons were identified at the same locations for *B. sinuspersici* and its close relative *B. cycloptera*.

At least one complete copy of the ycf1 gene was identified in the eight chloroplast genomes (total length of 5.3–5.6 Kb). In seven out of the eight chloroplast genomes, a duplicated ycf1 pseudogene (1,000–1,300 nt) was found at the IRa-SSC boundary. This is a common feature found in other species [23, 53]. In the case of *H. ammodendron*, there is a complete duplication of the ycf1 gene, therefore the *H. ammodendron* chloroplast genome has two full copies in the IR-SSC borders. The complete duplication of the ycf1 gene in *H. ammodendron* leads to the previously mentioned IR expansion (Additional file 4: Figure S3). This phenomenon has also been observed in *Amphilophium, Adenocalymma*, *Anemopaegma,* and *Fagopyrum* species; these species possess an expanded IR region and two full-length copies of ycf1 gene [23, 54, 55]. The IRs for the other seven species are variable in length. In *A retroflexus, B. muricata, B. cycloptera, B. sinuspersici, S. aralocaspica* and *S. maritima*, the IR includes the duplicated ycf1 pseudogene (1–1.3 kb) (Additional file 4: Figure S3). A small segment of the ycf1 gene is also duplicated in *V. vinifera, S. oleracea* and *B. vulgaris.* In *S. eltonica,* the IR has expanded to include the trnH-GTG and a fragment of the psbA gene (Additional file 4: Figure S3). The biological significance of this duplication remains unknown.

Annotation of the ycf15 gene with the Dual Organellar Genome Annotator (DOGMA) [44] shows variability in terms of its physical location. In *A. retroflexus, B. vulgaris* and *S. eltonica,* the ycf15 is located between the rps12 and trnV-GAC. In *B. cycloptera, B. muricata, B. sinuspersici, H. ammodendron, S. aralocaspica,* and *S. maritima* the ycf15 is located between ycf2 and trnL-CAA. The ycf15 as well as other genes, such as the ycF2, psbA, clpP, and matK, have been reported to have variable physical location in different plants [56–59].

The genes ycf3, clpP, rpoc1, and rpl2 have been found to have a variable number of introns among and within some taxonomic groups [23]. The gain or loss of introns in these genes have occurred independently in several linages of flowering plants [23, 60]. However, no differences were found in the number of introns among the eight species; the ycf3, clpP, rpoc1, and rpl2 contain 2, 2, 1, and 0 introns, respectively.

The orientation of the SSC region in *A. retroflexus, and B. muricata* differs from the orientation of the SSC in *B. cycloptera, B. sinuspersici, H. ammodendron, S. aralocaspica*, *S. maritima* and *S. eltonica* (Additional file 4: Figure S3). The SSC orientation has been shown to exist in the two different states within individual plants [61–64]. Therefore, SSC variation observed among taxa in this study is likely due to alternative states of the SSC region within individual plants. Although there was some variation in the SSC orientation, the number and content of genes was the same among the eight species. The only exception is the presence of a trnU-TCA in the SSC of *H. ammodendron*.

### Repeat structures and microsatellites

Seven out of the eight chloroplast genomes had 45–58 repeats, which ranged in length from 30 to 73 nt per repeat (Fig. 1). The majority of these repeats were shown to be between 30 and 40 nt in length. In the *S. eltonica* chloroplast genome, repeat analysis with REPuter [65] found a total of 174 repeats which ranged from 30 to 145 nt in length (Fig. 1). The number of repeats was similarly distributed among species for repeats found in intergenic regions and intron/exons (Table 4). An exception was *S. eltonica* in which a majority (80%) of repeats were located in the intergenic regions. Four species possessed reverse repeats; *S. maritima* and *S. aralocaspica* had one, *B. muricata* had two, and *S. eltonica* had four.

The presence of repeats varied for the genes ycf1, ycf2, ycf3, and psaA. Repeats were present in the gene ycf1 except for *A. retroflexus*, *S. aralocaspica* and *S. maritima*. All chloroplast genomes possessed repeats in the ycf2 gene except for *H. ammodendron*. Repeats in the introns of the ycf3 gene were only present in the *A. retroflexus, B. cycloptera*, and *B. sinuspersici*. All species presented at least one repeat in the psaA gene and *H. ammodendron* presented the highest number with six repeats.

Microsatellites, or simple sequence repeats (SSRs), were identified in the eight chloroplast genomes. The total number of microsatellites ranged from 41 to 72 of which the majority, 36–64, represent mononucleotide repeat microsatellites (Table 5). The complete list of microsatellites identified for each of the eight chloroplast genomes and their positions in the respective genomes is provided in Additional file 5: Table S2.

**Table 5:** Total number of microsatellites identified with MISA software for eight chloroplast genomes

Species name	Mono	Di	Tri	Compound	Total
*Amaranthus retroflexus*	44	2	0	5	51
*Bassia muricata*	40	1	0	1	42
*Bienertia cycloptera*	41	1	0	2	44
*Bienertia sinuspercisi*	48	0	0	3	51
*Haloxylon ammodendron*	46	0	0	2	48
*Suaeda aralocaspica*	47	3	0	1	51
*Suaeda eltonica*	64	2	1	5	72
*Suaeda maritima*	36	4	0	1	41

### Comparison of *Amaranthus retroflexus* chloroplast genome with previously sequenced *Amaranthus* spp. chloroplast genomes

*Amaranthus retroflexus*, commonly known as pigweed, is used as a vegetable for human consumption as well as for fodder. It is the most widely distributed and damaging *Amaranthus* weed in the US and the world [66]. Availability of the *A. retroflexus* chloroplast genome provides an important tool for accurately monitoring the spread of this species and identifying possible hybridizations. Microsatellites were previously identified for *Amaranthus* spp. [67]. Six out of the nine polymorphic microsatellites were shown to be polymorphic between A. *hypochondriacus* and *A. retroflexus* (Table 6). Most of these microsatellites were located in the LSC regions and represented A or T mononucleotide repeats. SSRs can serve as molecular markers for future molecular breeding for *Amaranthus* spp. which are considered as emerging crops [67]. The chloroplast genomes of four Amaranthus spp; *A.* *hypochondriacus, A. cruentus, A. caudatus,* and *A. hybridus*, have been reported previously [67]. The *A. hypochondriacus* genome (GenBank accession KX279888.1) is 150,725 nt and the quadripartite regions of LSC, SSC and 2 IRs consist of 83,873, 17,941 and 24,352 nts, respectively. These sizes are very similar to the lengths of the *A. retroflexus* chloroplast genome reported in this study (Table 2). BLAST analysis showed a 99% sequence similarity between the chloroplast genomes of *A. hypochondriacus* and *A. retroflexus*.

**Table 6:** Polymorphic simple sequence repeats (SSRs) in* Amaranthus hypochondriacus* and* A. retroflexus*

SRR location in *A. hypochondriacus* chloroplast genome (nt)	Repeat unit	Number of repeats	
		*A. hypochondriacus*	*A. retroflexus*
5,572–5,583	T	12	10
7,526–7,537	T	12	10
46,236–46,253	TA	9	8
46,573–46,588	AT	8	8
47,532–47,543	A	12	13
52,543–52,557	T	15	12
54,580–54,591	A	12	13
65,482–65,496	T	15	18
70,858–70,869	A	12	11
79,076–79,087	T	12	14
112,930–112,944	T	15	14
116,360–116,371	TATT	3	4

### Comparative analysis of the *B. sinuspersici* chloroplast genomes

Kim et al. [52], and Caburatan et al. [68] previously reported the chloroplast genome of *B. sinuspersici* (GenBank accession no. KU726550). Compared to our results with *B. sinuspersici* (Table 2), the size of their genome (153,472 nt) is 138 nt larger; the LSC and SSC in their study are 84,560 nt and 19,016 nt in size, respectively which is 70 nt larger than in our study (Table 2). The IR was reported to be 24,948 nt in length, versus 24,949 nt length in this study. The increase in length in the published *B. sinuspersici* chloroplast genome [52] is predominantly located at the LSC-IRa and SSC-IRb junctions, which has a repeat of 72 and 13 nts respectively. The two repeats are separated by spacer sequences of 1nt in the LSC-IRa junction and 48 nt in the SSC-IR junction. The 72 and 13 nt sequences were present just once in the *B. sinuspersici* chloroplast genome presented in the current study. The presence of a single occurrence of the 72 and 13 nt sequence in the genome was validated by Sanger sequencing of loci in question for both IRb-LSC and LSC-IRa loci (Additional file 2: Table S1). Further comparison of the two *B. sinuspersici* genomes identified 18 SNPs and 9 indels. In the published *B. sinuspersici* chloroplast genome, the LSC is inverted with respect to the rest of the sequence (IRa + SSC + IRb). In our study, the orientation of the LSC was validated using Sanger sequencing of PCR amplicons spanning the junctions IRb-LSC and LSC-IRa (Additional file 2: Table S1). As described above, there were also differences in the presence of stop codons in the rpl23 gene. In the previous study [68] a total of 110 unique genes were reported; a total of a total of 114 genes were identified in the current study (Additional file 4: Figure S3).

Differences between the previously reported chloroplast genome of *B. sinuspersici* compared to the current study likely stems from how the Celera assembler algorithm and the CLC algorithm process the read data. Each of these algorithms have their inherent pros and cons [69]. The assembly parameters for the previous *B. sinuspersici* chloroplast genome were not reported. Also, the chloroplast genome loci that were found to be different within the two previous versions [52, 68] were not resequenced. The chloroplast genome of *B. sinuspersici* presented in this study showed a minimum, maximum and average coverage of 37, 23,533, 3,204.28 nt. Furthermore, areas of ambiguity were validated via Sanger sequencing of PCR amplicons generated from selected loci. The combination of the assembly strategy utilized, and resequencing of loci, resulted in the generation of an improved version of the *B. sinuspersici* chloroplast genome.

Analysis of the two closest SCC_4_ related species, *B. cycloptera* and *B. sinuspersici*, chloroplast genomes showed a 99.70% sequence similarity between both sequences. *B. cycloptera* and *B. sinuspersici* chloroplast genomes differed in overall length by seven nt. *B. sinuspersici* IR, and SSC regions were larger than the *B. cycloptera* by 44 nt and *B. cycloptera*’s LSC region was larger by 51 nt. The difference in size was due to changes in the intergenic region, length, and number of repeat regions. Number of genes with introns and repeats was the same between the two species. *B. cycloptera* had two larger repeats, one between 40–44 nt and the second greater than 45 nt. *B. sinuspersici* had one smaller repeat of 30–34 nt. Both species had the same number and identity of protein-coding, tRNA, and rRNA genes.

### Comparative analysis of *Haloxylon ammodendron* chloroplast genomes: a case of transfer of mitochondrial DNA to the plastid genome

The chloroplast genome of *H. ammodendron* was published recently (GenBank accession no. KF534478) [70]. The size of the chloroplast genome was reported to be 151,570 nt, with a LSC of 84,214 nt, SSC of 19,014 nt and two IRs of 24,171 nt [70]. In our study, the genome assembled to a size of 161,251 nt, which is 9,681 nts larger. BLAST alignment of the two genomes indicated that the additional 9,681 nts were derived from the expansion of the IR, which is 4,868 nt in size. The IRs of *H. ammodendron* chloroplast genome in our study were 29,061 nt long. This represents an expansion of the IR that is also observed in *S. eltonica* (Table 2). Expansion and gene duplication are common phenomenon in the IR regions of chloroplast genomes [71, 72]. In grasses, the junctions between the IR and SSC regions are highly variable with the ends of genes ndhF, rps19, and ndhH repeatedly migrating into and out of the adjacent IR regions [73]. BLAST alignment between the two genomes revealed that the first 115 nt showed 78% homology with chloroplast sequences of *H. persicum*, and *H. ammodendron* present in the IRs of the published genomes [70]. The following region of 671 nt did not show any significant similarity and the last 4,028 nt showed homology to mitochondrial genome sequences. The highest significant hit (94%; E value = 0.0) for this 4,028 nt section resembled *Beta vulgaris* and *Spinacia oleraceae*. Interestingly, annotation identified the mitochondrial gene Cytochrome b (cob) in this 4,814 nt section, although the plastid copy had a nonsense mutation that resulted in a premature stop codon.

Evidence showing transfer of mitochondrial DNA (mtDNA) or nuclear DNA (nucDNA) to the plastid genome in plants had been lacking until recently. A few recent reports indicate that plastid genomes of carrot [74], milkweed [75], and bamboo [73] show evidence of gene transfer from mitochondria to the plastid. *Daucus carota* has a 1.5 kb region of mitochondrial origin located in the rps12-trnV intergenic space of the chloroplast genome. Only *Daucus* species and the close relative *Cuminum cyminum* (cumin) show the mitochondrion-to-chloroplast gene transfer [74]. It was concluded that a mitochondria-located DNA segment present in the ancestor of the Apiaceae subsequently moved to the plastid genome in the common ancestor of *Daucus* and cumin. *Asclepias syriaca,* the common milkweed, has a 2.4 kb mtDNA-like insert in the chloroplast genome. The mtDNA-like insert contains an intact exon of the mitochondrial ribosomal protein (rpl2) as well as a noncoding region [75]. There was a 92% sequence identity between the mitochondrial and plastid version of rpl2 in *A. syriaca* whereas the plastid copy had a nonsense mutation resulting in a premature stop codon. Similarly, the IR region in three herbaceous bamboo species of the *Pariana* genus had a 2.7 kb insertion [73]. The insertion was located in the trnI-CAU-trnL-CAA intergenic spacer region. Potential variations of this insertion in another *Pariana* species and species from the sister genus *Eremitis* were also reported*.* These studies suggest that the transferred sequence may have originated as a single event in a common ancestor; however, the inserted sequence evolved rapidly [73].

In our study, the inserted section in *H. ammodendron* had an average coverage of 1,320X reported from the stringent 0.99–0.99 length fraction/similarity mapped to the assembly. The coverage corresponded well to the average coverage of 1,269X for other regions. Five kb regions flanking the 4.8 kb section had a similar coverage of 929 and 1,066 reads. The Illumina reads from *H. ammodendron* (0.99–0.99 99 length fraction/similarity fraction) were mapped to three randomly selected intronless mitochondrial genes identified from the *H. ammodendron* assembly [73]. The mitochondrial genes ccmFN, matR and rrn26 showed a much lower average coverage of 242, 211, and 447, respectively. Thus, the mapping results supported the result that the insertion in the *H. ammodendron* chloroplast genome was not an artifact of the assembly.

**Fig. 2:**
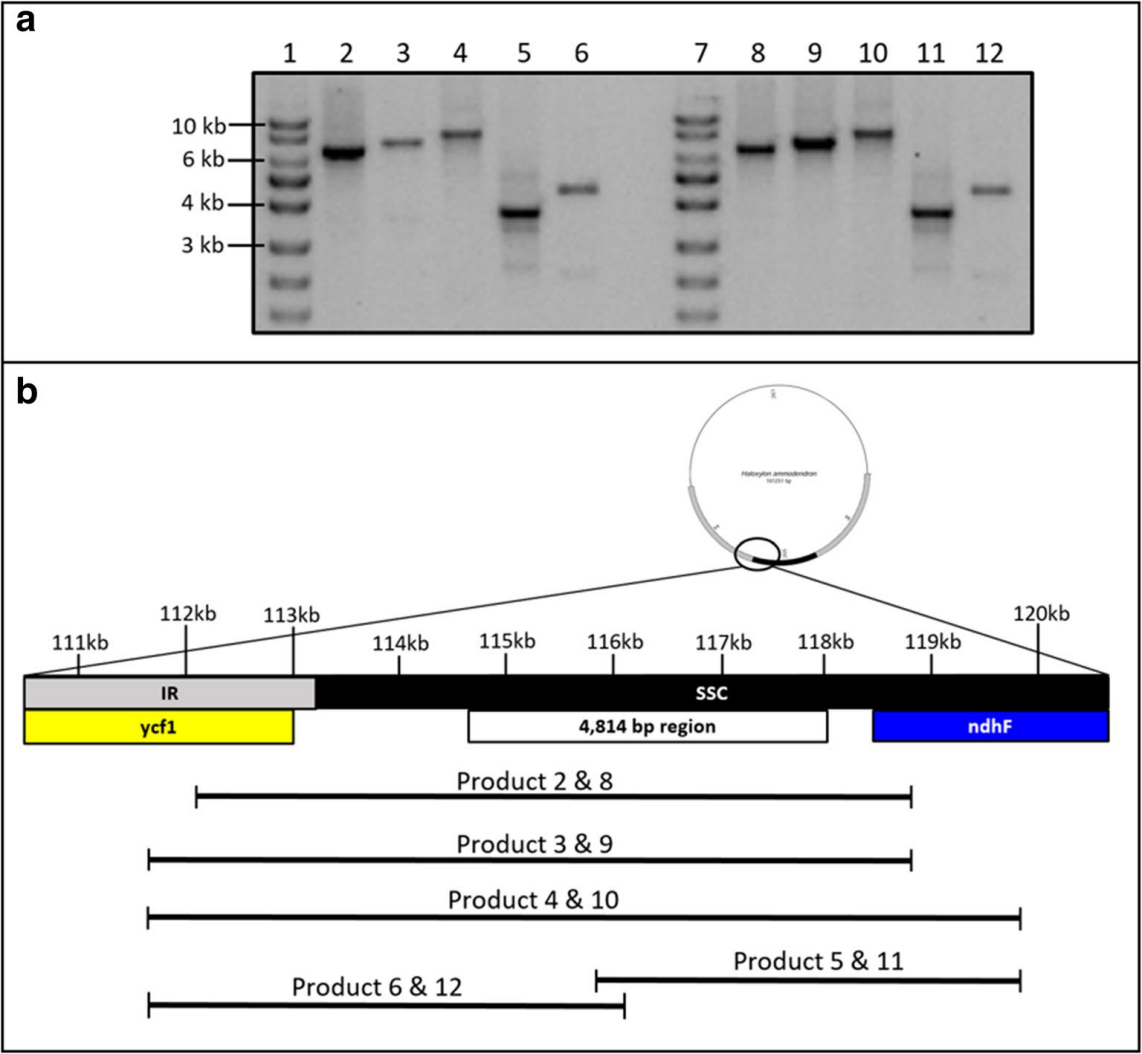
PCR amplicons flanking a 4.8 kb insertion in the chloroplast genome of *H. ammodendron* (2–6) and *H. persicum* (8–12). Agarose gel electrophoresis of PCR products (**a**); diagram representing location and length of each amplicon (**b**). Expected amplicon sizes are 6607 (2 and 8), 7172 (3 and 9), 8132 (4 and 6), 3810 (5 and 11), and 4458 nt (6 and 12). Primers for PCRs 2–4 and 8–10 flank the ycf1 and ndhF genes. Primers for PCRs 5 and 11 flank flank the middle section of the 4.8 kb insertion and the ndhF gene. Primers for PCRs 6 and 12 flank the ycf1 gene and the middle section of the 4.8 kb insertion. 1 and 7: exACTGene DNA Ladders 1 kb DNA Ladder

Since the *H. ammodendron* chloroplast genome reported in this study was assembled from reads obtained using total cellular DNA, the origin of 4.8 kb insert was confirmed using a complementary Sanger sequencing approach. Amplified segments flanking the entire 4,814 nt insertion were 6,607, 7,172 and 8,132 nt long with the forward and the reverse primers flanking the ycf1 and ndhF genes, respectively (Fig. 2; Additional file 6: Table S3). Primers flanking both the ycf1 and ndhF genes coupled with a primer annealing to the middle section of the inserted region produced amplicons of predicted sizes of 3,810 and 4,458 nt (Fig. 2; Additional file 6: Table S3). The PCR results were the first line of confirmation since no PCR amplification should be expected from the published *H. ammodendron* chloroplast genome due to primer mismatch. Interestingly, expected DNA amplicons were also obtained when PCR was performed on *Haloxylon persicum*, a close relative of *H. ammodendron* (Fig. 2). A total section of 6.2 kb, including the 4,814nt inserted section, was sequenced and validated via primer walking (Additional file 6: Table S3). The sequenced amplicon results produced a 100% alignment match to the *H. ammodendron* chloroplast genome assembly obtained in this study. Amplification and sequence homology validation of the 4,814 nt section confirmed the presence of the insertion in the *H. ammodendron* chloroplast genome. The integration of intracellularly transferred DNA into the intergenic region of ycf1 and ndhF would be expected as insertion in the coding region would have disrupted gene function.

This is the first report to document mitochondria-to-chloroplast interorganellar gene transfer in the Chenopodiaceae family and the fourth example in angiosperms. However, the mechanisms underlying the transfer of genomic DNA fragments remains to be elucidated [73–75].

### Chloroplast genomes among different types of C_4_ species versus C_3_ species

The 8 chloroplast genomes studied, include the C_3_ species *S. maritima* and 7 forms of C_4_ species. The results indicate the chloroplast genomes are very similar in the number (82–84) and type of CDS genes encoding proteins. Despite some differences in gene content and organization among the chloroplast genomes, these differences do not coincide with the type of oxygenic photosynthesis (C3 or C4) that these 8 species represent. There is a general conservation of genes present in the C_3_ species *B. muricata* and the C_4_ species. This suggests nuclear genes encode most chloroplast-targeted proteins that are needed to support the C_4_ pathway. Both Kranz type and single-cell type C_4_ species have dimorphic chloroplasts (relative to function in carbon assimilation, starch synthesis, and in relative expression of photosystem I and photosystem II for balancing requirements for ATP and NADPH). In carbon assimilation one type of chloroplast supports fixation of atmospheric CO_2_ by PEPC with synthesis of C_4_ acids. They generate energy to support conversion of pyruvate to phosphoenolpyruvate utilizing pyruvate, P*i* dikinase, adenylate kinase, and inorganic pyrophosphatase, and they support reduction of oxaloacetate to malate by NADP-malate dehydrogenase. The other type of chloroplast has the Calvin-Benson cycle with Rubisco fixing CO_2_ that is generated by decarboxylation of C_4_ acids (utilizing plastid-targeted NADP-malic enzyme in some C_4_ species). Currently all enzymes required in chloroplasts to support the C_4_ cycle and Calvin-Benson cycle are considered to be nuclear encoded except the gene for the large subunit of Rubisco which is in the chloroplast genome, while the small subunit gene is in the nucleus [39, 76–79]. In the dual-cell Kranz type C_4_ plants, cell specific control of transcription of nuclear genes may contribute to development of dimorphic chloroplasts. Other mechanisms must control development of dimorphic chloroplasts in SCC_4_ species (see hypotheses, selective protein import, selective mRNA targeting, selective protein degradation; [77]). Future studies are needed to determine how dimorphic chloroplasts develop to coordinate function of C_4_ in carbon assimilation, metabolite transport between chloroplasts, and requirements of energy from photochemistry.

## Conclusions

This study reports high quality, and complete chloroplast genomes from seven Chenopodiaceae and one Amaranthaceae species. The procedures show the hybrid method of using high throughput and Sanger sequencing [80, 81] is rapid, efficient, and reliable for chloroplast genome sequencing. While genome organization, gene order, and content were largely conserved, there were a few structural differences, such as the variable location of the ycf15 gene; the high repeat content in the *S. eltonica* genome; the presence of two copies of ycf1 gene in *H. ammodendron* along with the IR expansion; and the IR expansion in *S. eltonica* that includes the trnH-GTG and psbA. The biological significance of these differences remains to be investigated.

The *B. sinuspersici* chloroplast genome presented in this study represents an improved version due to the high sequencing coverage and the validation of the junction regions through Sanger sequencing. The improvement in the *B. sinuspersici* chloroplast genome sequence allowed for the identification of a higher number of chloroplast genes. Interestingly, the *H. ammodendron* chloroplast genome presented in this study is 9,681 nt larger than the previously published genome [70]. This difference originated from a duplicated region of the IR, which is 4,868 nt in size and represented a rare instance of interorganellar DNA transfer from the mitochondria to the chloroplast genome.

The purpose of this study was to analyze chloroplast genomes in a few representative dicot species which have different forms of photosynthesis. Due to the high number of variable photosynthetic types present in Chenopodiaceae and almost 90% of the gene products in the chloroplast originating in the nucleus, there may be an expectation that the Chenopodiaceae may include chloroplast-encoded genes corresponding to each photosynthetic phenotype. However, to derive such phylogenetic conclusions requires extensive taxon sampling as exemplified in a recent analysis of 113 grass species [82]. Therefore, such an analysis was outside the purview of the current study.

C_4_ plants evolved independently from C_3_ species more than 60 times [33] leading to development of different forms of Kranz, along with single-cell C_4_ species, all of which have dimorphic chloroplasts coordinated in functions to support C_4_ photosynthesis. This includes differential expression of enzymes in carbon assimilation, selective expression of metabolite transporters to control flux of carbon between the two chloroplasts, and expression of photosystem I and II for production of ATP and NADPH. How these dimorphic chloroplasts develop through control of expression of nuclear and chloroplast genes remains unknown. Complete chloroplast genomic information on different forms of C_4_ species across dicot and monocot families should be useful in future studies on the control of its development, determining what is required for C_4_ photosynthesis, and determining the degree of conservation of the chloroplast genome in these photosynthetic types across phylogeny.

## Materials and methods

### Plant material and DNA extraction

*Amaranthus retroflexus, Bassia muricata, Suaeda eltonica* and *Suaeda maritima* plants were grown in a growth chamber with a 14/10 h photoperiod, light regime of 525 PPFD and day/night, and temperature of 28 °C/18 °C. The same photoperiod and light regime were used for *Bienertia cycloptera*, *B. sinuspersici* and *Suaeda aralocaspica*; however, the day/night temperatures were modified to 35 °C/18 °C. *Haloxylon ammodendron* plants were grown under natural annual environmental conditions in Pullman, WA. Total cellular DNA was isolated using fresh leaf tissue from each species with a Urea Lysis Buffer Method. Briefly, leaf tissue was flash frozen in liquid nitrogen and ground to a fine powder and approximately 100 mg tissue was placed in 600 μL buffer containing 42% w/v Urea, 250 mM NaCl, 50 mM Tris (pH 8.0), 1% sodium dodecyl sulfate (SDS) and 20 mM EDTA. Solution was briefly vortexed, extracted with equal volume of 1:1 phenol: chloroform and vortexed for 45 s. Samples were then centrifuged at 9,500 x *g* for 5 min and the supernatant was added to an equal volume of ice cold 2-propanol. The tube was rocked gently six times and centrifuged for 10 min at 9,500 x *g*. The pellet was washed in 1 mL ice cold 70% ethanol and centrifuged at 9,500 x *g* for 2 min and the supernatant was decanted. The pellet was dried and suspended in 500 μL TE buffer with 20 μg/mL RNAse A and incubated for 30 min at 37 °C prior to the addition of 1/10th volume 3 M sodium acetate (pH 5.3) and 2 volumes of 95% ethanol and rocked gently 6 times. The tube was centrifuged at 9,500 x *g* for 10 min, supernatant removed and the pellet was rinsed with 500 μL 70% ethanol, centrifuged for 2 min at 9,500 x *g* and the pellet was dried before being suspended in 50 μL TE buffer.

### DNA sequencing, validation and contig assembly

The paired-end DNA sample prep kit (PE-102–1001; Illumina, San Diego, CA) was used to generate a paired-end library according to manufacturer’s recommendations (Illumina, San Diego, CA) at the Research Technology Support Facility at Michigan State University (East Lansing, MI, USA). DNA samples were sequenced on the Illumina HiSeq 2000 utilizing the 100PE chemistry. Quality control on raw sequence data was performed using CLC Genomics Workbench ver. 6.0.1 (CLC), (QIAGEN, Redwood City, CA, USA). CLC was utilized for read trimming, merging reads and filtering out low quality sequences with a phred score below 40. Assembly and mapping of the reads to the contigs was accomplished with CLC software. Mapping of reads to contigs was conducted using the following mapping parameters: mismatch cost 2, insertion cost 3, deletion cost 3, length fraction 0.8 and similarity fraction 0.9. BLASTN searches on NCBI (https://www.ncbi.nlm.nih.gov/) were performed using the assembled contigs as query sequences to identify contigs with high homology to chloroplast large single copy (LSC), small single copy (SSC) and inverted repeat (IR) for each of the assembled libraries obtained from each of the eight plant species. Identified IR contigs were reverse complimented and overlapping borders of each of the identified contigs were aligned to assemble a complete chloroplast genome sequence in the following order of LSC + IR + SSC + IR. Chloroplast contig junctions from overlapping border regions were aligned and analyzed with MEGA6 version 6.0.6 (https://www.megasoftware.net/). Flanking primers for chloroplast junctions were designed utilizing Primer3 Software [83]. PCR amplification was performed using Platinum Taq High-Fidelity DNA polymerase (Invitrogen, CA) and PCR products were purified using the QIAquick PCR purification Kit (QIAGEN, MD). Amplicons, ranging in size from 0.2 to 0.5 kb, were Sanger sequenced to ensure sequence fidelity of the DNA assembly output (Eurofins Genomics, KY). A primer walking and Sanger sequencing method was utilized to identify non-overlapping regions in the LSC + IRa and IRb + LSC junctions of *T. indica* and the IRa + SSC and SSC + IRb junctions of *S. eltonica.* The primer walking and Sanger sequencing method was also employed to validate specific conflicting sequences in the *H. ammodendron* chloroplast genome when compared to the publicly available *H. ammodendron* sequence. A remapping of the Illumina sequenced reads was performed using the final predicted chloroplast genomes from the eight species utilizing CLC software. A length fraction and similarity fraction of 0.99 were chosen as remapping parameters to ensure high stringency alignment. Assemblies generated with 0.80–0.90 and 0.99–0.99 length fraction and similarity fraction were screened to identify regions with coverage below 40 ×. Sequence data have been deposited to GenBank database under accession numbers MT299584 (*A. retroflexus*), MT316306 (*B. muricata*), MT316305 (*B. cycloptera*), MT316307 (*B. sinuspersici*), MT316308 (*H. ammodendron*), MT316309 (*S. aralocaspica*), MT316310 (*S. eltonica*), and MT316311 (*S. maritima*).

### Genome annotation and visualization

All the chloroplast genomes were annotated and visualized with GeSeq [43] which incorporates the Dual Organellar Genome Annotator (DOGMA) [44] and OrganellarGenomeDRAW (OGDRAW) [84].

### Comparisons of gene content and gene order

Comparisons for both gene content and order were performed for the eight chloroplast sequences. This comparison included three chloroplast reference genomes: *V. vinifera* (NC_007957.1)*, S. oleracea* (AJ400848.1) and *B. vulgaris* (EF534108.1). Gene order and content were parsed manually using pair-wise comparisons between species.

### Examination of repeat structure and microsatellites

REPuter [65] was utilized to identify the number and location of forward, reverse, complementary, and palindromic repeats in the sequence of the eight species predicted chloroplast sequences. A minimum repeat size of 30 nt and a Hamming distance of 3 (> 90% sequence identity) was utilized. Shared and unique repeats were identified manually and with the use of BLASTN based on intergenomic comparisons.

Microsatellites were identified with MISA software [85] using standard thresholds. Specifically, a minimum stretch of 10 for mono-, six for di-, five for tri-, and three for tetra-, penta-, and hexa-nucleotide repeats, and a minimum distance of 100 nucleotides between compound microsatellites.

## Supplementary information

**Additional file 1: Figure S1.** Representative example of an overlap region amplicon sequenced with Sanger approch. The IRA-SSC junction showed a 100% match during nucleotide alignment.

**Additional file 2: Table S1.** Forward and reverse primers used to amplify and validate the overlapping regions present in all four possible junctions (LSC-IR, IR-SSC, SSC-IR, and IR-LSC) of eight chloroplast genomes.

**Additional file 3: Figure S2.** Stack column graphs of minimum coverage (MC) and average coverage (AC) for eight chloroplast genomes assembled with 80%-90% (blue) and 99%-99% (orange) length fraction-similarity fraction parameters.

**Additional file 4: Figure S3.** Representative maps of the chloroplast genome of A. *Amaranthus retroflexus*, B. *Bassia muricata*, C. *Bienertia cycloptera*, D. *B. sinuspersici*, E. *Haloxylon ammodendron*, F. *Suaeda aralocaspica*, G. *S. eltonica*, and H. *S. maritima*. Genes shown outside the outer circle are transcribed clockwise whereas those represented inside are transcribed counterclockwise. Large single copy (LSC), small single copy (SSC), and inverted repeats (IRa, IRb) regions are indicated.

**Additional file 5: Table S2.** Forward and reverse primers used to amplify and validate the mitochondrion-to-plastidial DNA transfer in *Haloxylon ammodendron* and *H. persicum*.

**Additional file 6:** Forward and reverse primers used to amplify and validate the mitochondrion-to-plastidial DNA transfer in *Haloxylon ammodendron* and *H. persicum*.

## Data Availability

The sequence of the chloroplast genomes generated during the current study are accessible from GenBank accession numbers: MT299584 (*A. retroflexus*), MT316306 (*B. muricata*), MT316305 (*B. cycloptera*), MT316307 (*B. sinuspersici*), MT316308 (*H. ammodendron*), MT316309 (*S. aralocaspica*), MT316310 (*S. eltonica*), and MT316311 (*S. maritima*), (https://www.ncbi.nlm.nih.gov/genbank/).
