# Supplementary material for: Methods of analysis of chloroplast genomes of C3, Kranz type C4 and Single Cell C4 photosynthetic members of Chenopodiaceae
Source: Plant Methods. 2020 Aug 31;16:119. doi: 10.1186/s13007-020-00662-w (PMC7457496; doi:10.1186/s13007-020-00662-w)
Supplement: Supplementary file 1 — Additional file 1: Figure S1. Representative example of an overlap region amplicon sequenced with Sanger approch. The IRA-SSC junction showed a 100% match during nucleotide alignment. [file 13007_2020_662_MOESM1_ESM.pptx]

## Slide 1
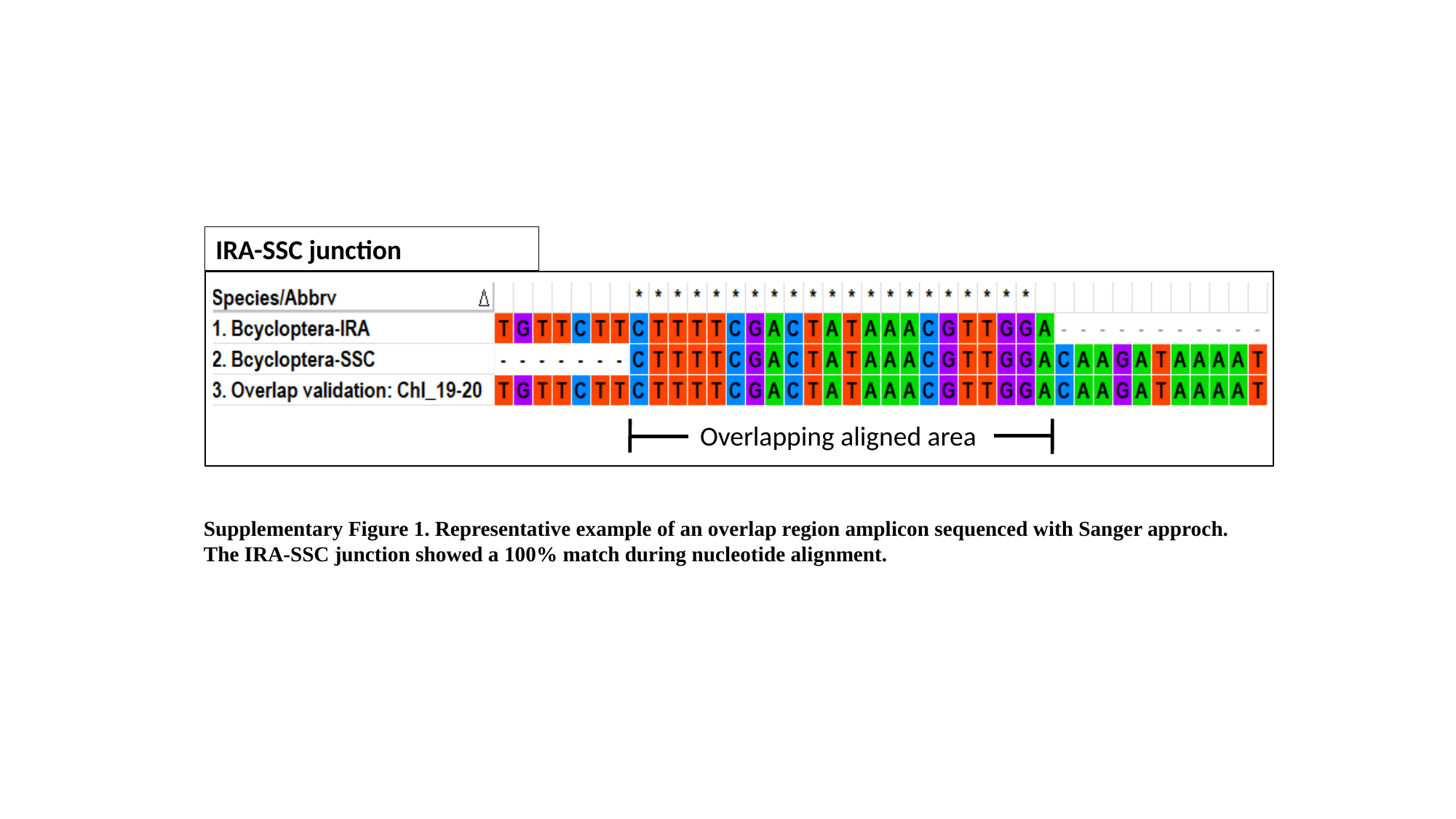

IRA-SSC junction
Overlapping aligned area
Supplementary Figure 1. Representative example of an overlap region amplicon sequenced with Sanger approch. The IRA-SSC junction showed a 100% match during nucleotide alignment.
