# Supplementary material for: Methods of analysis of chloroplast genomes of C3, Kranz type C4 and Single Cell C4 photosynthetic members of Chenopodiaceae
Source: Plant Methods. 2020 Aug 31;16:119. doi: 10.1186/s13007-020-00662-w (PMC7457496; doi:10.1186/s13007-020-00662-w)
Supplement: Supplementary file 2 — Additional file 2: Table S1. Forward and reverse primers used to amplify and validate the overlapping regions present in all four possible junctions (LSC-IR, IR-SSC, SSC-IR, and IR-LSC) of eight chloroplast genomes. [file 13007_2020_662_MOESM2_ESM.pptx]

## Slide 1
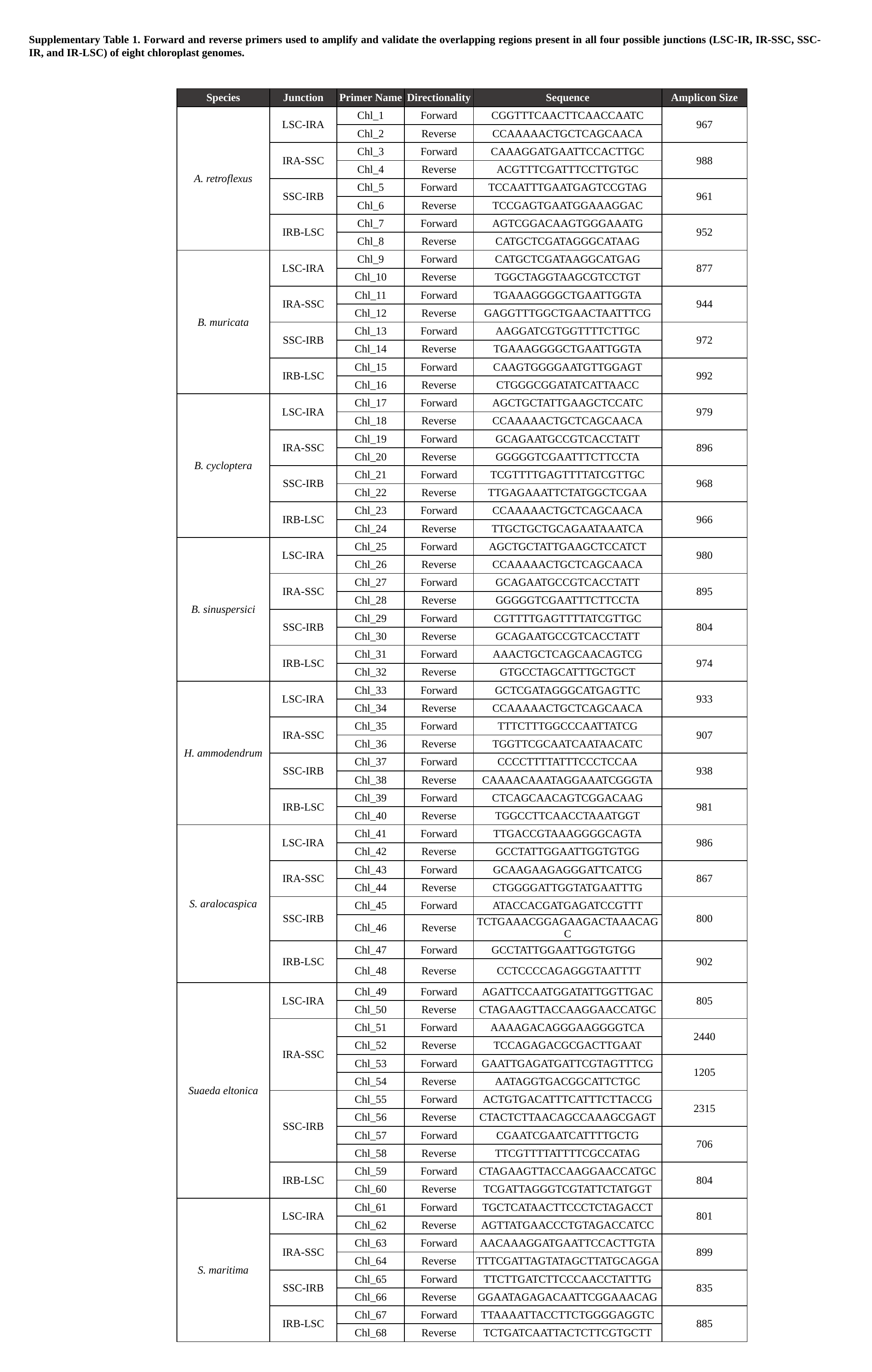

Supplementary Table 1. Forward and reverse primers used to amplify and validate the overlapping regions present in all four possible junctions (LSC-IR, IR-SSC, SSC-IR, and IR-LSC) of eight chloroplast genomes.
| Species | Junction | Primer Name | Directionality | Sequence | Amplicon Size |
| --- | --- | --- | --- | --- | --- |
| A. retroflexus | LSC-IRA | Chl\_1 | Forward | CGGTTTCAACTTCAACCAATC | 967 |
| | | Chl\_2 | Reverse | CCAAAAACTGCTCAGCAACA | |
| | IRA-SSC | Chl\_3 | Forward | CAAAGGATGAATTCCACTTGC | 988 |
| | | Chl\_4 | Reverse | ACGTTTCGATTTCCTTGTGC | |
| | SSC-IRB | Chl\_5 | Forward | TCCAATTTGAATGAGTCCGTAG | 961 |
| | | Chl\_6 | Reverse | TCCGAGTGAATGGAAAGGAC | |
| | IRB-LSC | Chl\_7 | Forward | AGTCGGACAAGTGGGAAATG | 952 |
| | | Chl\_8 | Reverse | CATGCTCGATAGGGCATAAG | |
| B. muricata | LSC-IRA | Chl\_9 | Forward | CATGCTCGATAAGGCATGAG | 877 |
| | | Chl\_10 | Reverse | TGGCTAGGTAAGCGTCCTGT | |
| | IRA-SSC | Chl\_11 | Forward | TGAAAGGGGCTGAATTGGTA | 944 |
| | | Chl\_12 | Reverse | GAGGTTTGGCTGAACTAATTTCG | |
| | SSC-IRB | Chl\_13 | Forward | AAGGATCGTGGTTTTCTTGC | 972 |
| | | Chl\_14 | Reverse | TGAAAGGGGCTGAATTGGTA | |
| | IRB-LSC | Chl\_15 | Forward | CAAGTGGGGAATGTTGGAGT | 992 |
| | | Chl\_16 | Reverse | CTGGGCGGATATCATTAACC | |
| B. cycloptera | LSC-IRA | Chl\_17 | Forward | AGCTGCTATTGAAGCTCCATC | 979 |
| | | Chl\_18 | Reverse | CCAAAAACTGCTCAGCAACA | |
| | IRA-SSC | Chl\_19 | Forward | GCAGAATGCCGTCACCTATT | 896 |
| | | Chl\_20 | Reverse | GGGGGTCGAATTTCTTCCTA | |
| | SSC-IRB | Chl\_21 | Forward | TCGTTTTGAGTTTTATCGTTGC | 968 |
| | | Chl\_22 | Reverse | TTGAGAAATTCTATGGCTCGAA | |
| | IRB-LSC | Chl\_23 | Forward | CCAAAAACTGCTCAGCAACA | 966 |
| | | Chl\_24 | Reverse | TTGCTGCTGCAGAATAAATCA | |
| B. sinuspersici | LSC-IRA | Chl\_25 | Forward | AGCTGCTATTGAAGCTCCATCT | 980 |
| | | Chl\_26 | Reverse | CCAAAAACTGCTCAGCAACA | |
| | IRA-SSC | Chl\_27 | Forward | GCAGAATGCCGTCACCTATT | 895 |
| | | Chl\_28 | Reverse | GGGGGTCGAATTTCTTCCTA | |
| | SSC-IRB | Chl\_29 | Forward | CGTTTTGAGTTTTATCGTTGC | 804 |
| | | Chl\_30 | Reverse | GCAGAATGCCGTCACCTATT | |
| | IRB-LSC | Chl\_31 | Forward | AAACTGCTCAGCAACAGTCG | 974 |
| | | Chl\_32 | Reverse | GTGCCTAGCATTTGCTGCT | |
| H. ammodendrum | LSC-IRA | Chl\_33 | Forward | GCTCGATAGGGCATGAGTTC | 933 |
| | | Chl\_34 | Reverse | CCAAAAACTGCTCAGCAACA | |
| | IRA-SSC | Chl\_35 | Forward | TTTCTTTGGCCCAATTATCG | 907 |
| | | Chl\_36 | Reverse | TGGTTCGCAATCAATAACATC | |
| | SSC-IRB | Chl\_37 | Forward | CCCCTTTTATTTCCCTCCAA | 938 |
| | | Chl\_38 | Reverse | CAAAACAAATAGGAAATCGGGTA | |
| | IRB-LSC | Chl\_39 | Forward | CTCAGCAACAGTCGGACAAG | 981 |
| | | Chl\_40 | Reverse | TGGCCTTCAACCTAAATGGT | |
| S. aralocaspica | LSC-IRA | Chl\_41 | Forward | TTGACCGTAAAGGGGCAGTA | 986 |
| | | Chl\_42 | Reverse | GCCTATTGGAATTGGTGTGG | |
| | IRA-SSC | Chl\_43 | Forward | GCAAGAAGAGGGATTCATCG | 867 |
| | | Chl\_44 | Reverse | CTGGGGATTGGTATGAATTTG | |
| | SSC-IRB | Chl\_45 | Forward | ATACCACGATGAGATCCGTTT | 800 |
| | | Chl\_46 | Reverse | TCTGAAACGGAGAAGACTAAACAGC | |
| | IRB-LSC | Chl\_47 | Forward | GCCTATTGGAATTGGTGTGG | 902 |
| | | Chl\_48 | Reverse | CCTCCCCAGAGGGTAATTTT | |
| Suaeda eltonica | LSC-IRA | Chl\_49 | Forward | AGATTCCAATGGATATTGGTTGAC | 805 |
| | | Chl\_50 | Reverse | CTAGAAGTTACCAAGGAACCATGC | |
| | IRA-SSC | Chl\_51 | Forward | AAAAGACAGGGAAGGGGTCA | 2440 |
| | | Chl\_52 | Reverse | TCCAGAGACGCGACTTGAAT | |
| | | Chl\_53 | Forward | GAATTGAGATGATTCGTAGTTTCG | 1205 |
| | | Chl\_54 | Reverse | AATAGGTGACGGCATTCTGC | |
| | SSC-IRB | Chl\_55 | Forward | ACTGTGACATTTCATTTCTTACCG | 2315 |
| | | Chl\_56 | Reverse | CTACTCTTAACAGCCAAAGCGAGT | |
| | | Chl\_57 | Forward | CGAATCGAATCATTTTGCTG | 706 |
| | | Chl\_58 | Reverse | TTCGTTTTATTTTCGCCATAG | |
| | IRB-LSC | Chl\_59 | Forward | CTAGAAGTTACCAAGGAACCATGC | 804 |
| | | Chl\_60 | Reverse | TCGATTAGGGTCGTATTCTATGGT | |
| S. maritima | LSC-IRA | Chl\_61 | Forward | TGCTCATAACTTCCCTCTAGACCT | 801 |
| | | Chl\_62 | Reverse | AGTTATGAACCCTGTAGACCATCC | |
| | IRA-SSC | Chl\_63 | Forward | AACAAAGGATGAATTCCACTTGTA | 899 |
| | | Chl\_64 | Reverse | TTTCGATTAGTATAGCTTATGCAGGA | |
| | SSC-IRB | Chl\_65 | Forward | TTCTTGATCTTCCCAACCTATTTG | 835 |
| | | Chl\_66 | Reverse | GGAATAGAGACAATTCGGAAACAG | |
| | IRB-LSC | Chl\_67 | Forward | TTAAAATTACCTTCTGGGGAGGTC | 885 |
| | | Chl\_68 | Reverse | TCTGATCAATTACTCTTCGTGCTT | |
