# Supplementary material for: Methods of analysis of chloroplast genomes of C3, Kranz type C4 and Single Cell C4 photosynthetic members of Chenopodiaceae
Source: Plant Methods. 2020 Aug 31;16:119. doi: 10.1186/s13007-020-00662-w (PMC7457496; doi:10.1186/s13007-020-00662-w)
Supplement: Supplementary file 3 — Additional file 3: Figure S2. Stack column graphs of minimum coverage (MC) and average coverage (AC) for eight chloroplast genomes assembled with 80%-90% (blue) and 99%-99% (orange) length fraction-similarity fraction parameters. [file 13007_2020_662_MOESM3_ESM.pptx]

## Slide 1
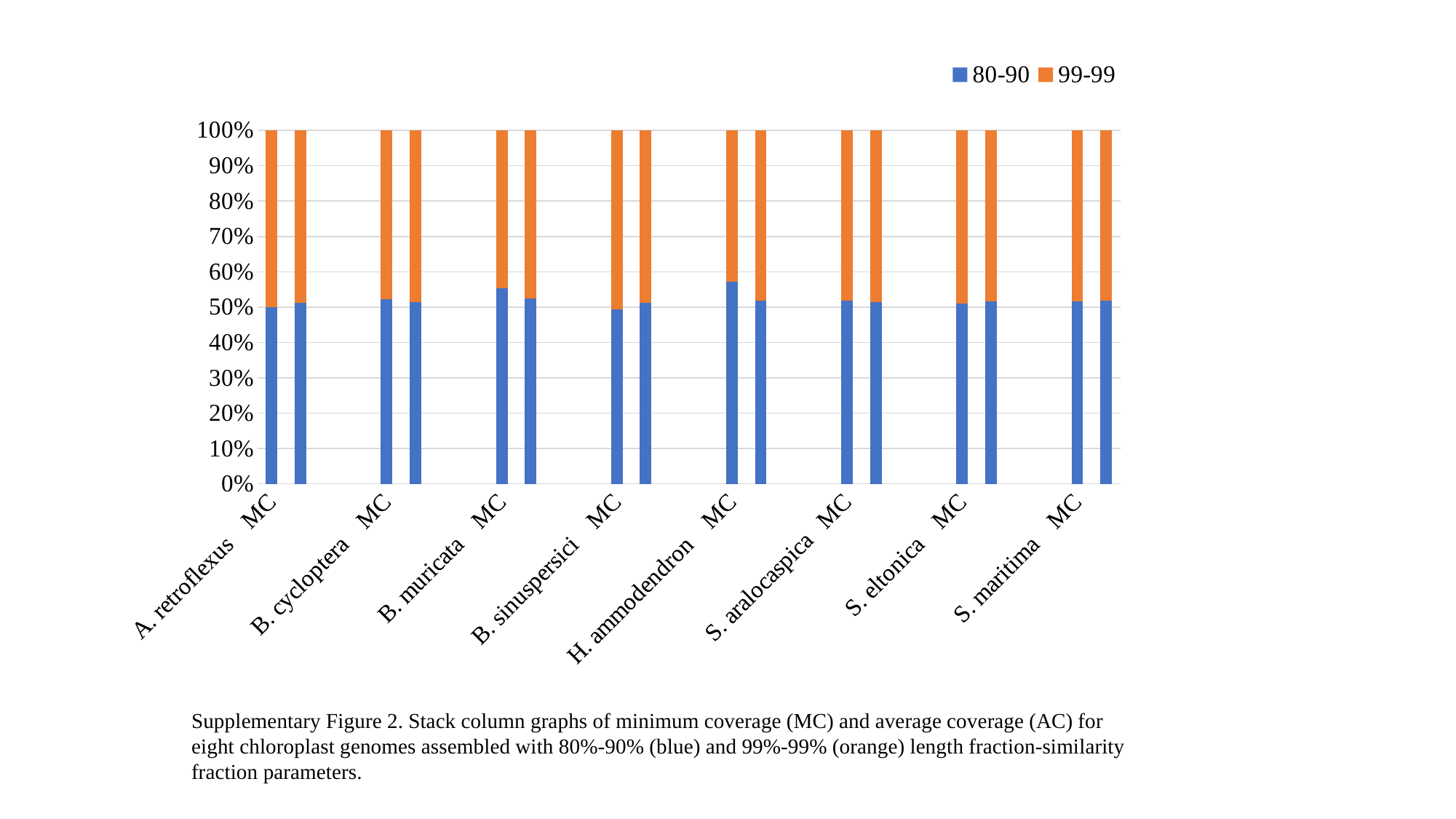

### Chart
| Category | 80-90 | 99-99 |
|---|---|---|
| A. retroflexus MC | 22.0 | 22.0 |
| AC | 3649.67 | 3476.6 |
| | None | None |
| | None | None |
| B. cycloptera MC | 12.0 | 11.0 |
| AC | 1553.12 | 1468.5 |
| | None | None |
| | None | None |
| B. muricata MC | 37.0 | 30.0 |
| AC | 3204.28 | 2901.98 |
| | None | None |
| | None | None |
| B. sinuspersici MC | 39.0 | 40.0 |
| AC | 5998.08 | 5710.16 |
| | None | None |
| | None | None |
| H. ammodendron MC | 12.0 | 9.0 |
| AC | 1357.2 | 1259.62 |
| | None | None |
| | None | None |
| S. aralocaspica MC | 55.0 | 51.0 |
| AC | 4864.44 | 4606.14 |
| | None | None |
| | None | None |
| S. eltonica MC | 25.0 | 24.0 |
| AC | 1591.56 | 1486.77 |
| | None | None |
| | None | None |
| S. maritima MC | 33.0 | 31.0 |
| AC | 4111.85 | 3832.41 |Supplementary Figure 2. Stack column graphs of minimum coverage (MC) and average coverage (AC) for eight chloroplast genomes assembled with 80%-90% (blue) and 99%-99% (orange) length fraction-similarity fraction parameters.
