# Supplementary material for: Methods of analysis of chloroplast genomes of C3, Kranz type C4 and Single Cell C4 photosynthetic members of Chenopodiaceae
Source: Plant Methods. 2020 Aug 31;16:119. doi: 10.1186/s13007-020-00662-w (PMC7457496; doi:10.1186/s13007-020-00662-w)
Supplement: Supplementary file 4 — Additional file 4: Figure S3. Representative maps of the chloroplast genome of A. Amaranthus retroflexus, B. Bassia muricata, C. Bienertia cycloptera, D. B. sinuspersici, E. Haloxylon ammodendron, F. Suaeda aralocaspica, G. S. eltonica, and H. S. maritima. Genes shown outside the outer circle are transcribed clockwise whereas those represented inside are transcribed counterclockwise. Large single copy (LSC), small single copy (SSC), and inverted repeats (IRa, IRb) regions are indicated. [file 13007_2020_662_MOESM4_ESM.pptx]

## Slide 1
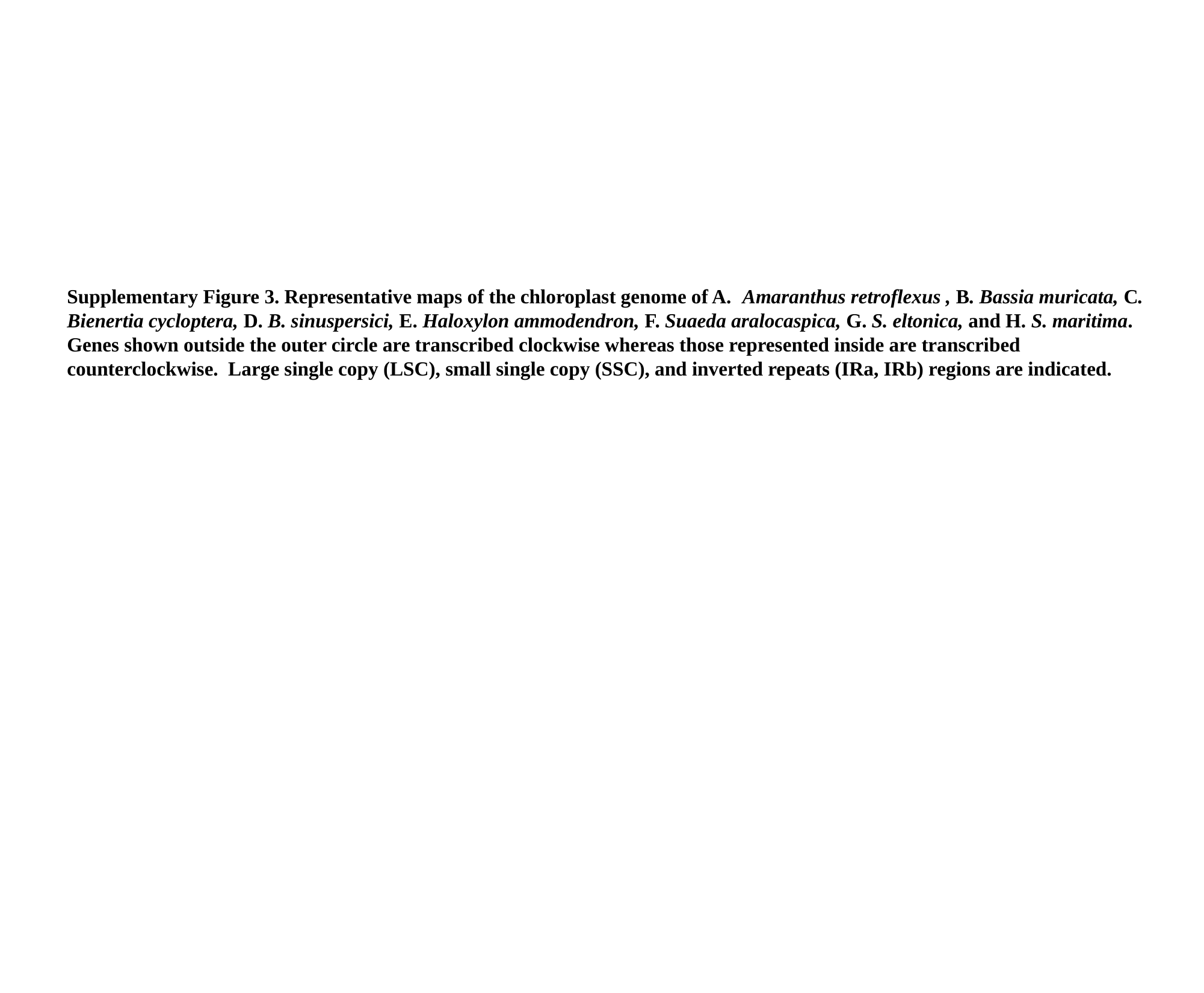

Supplementary Figure 3. Representative maps of the chloroplast genome of A. Amaranthus retroflexus , B. Bassia muricata, C. Bienertia cycloptera, D. B. sinuspersici, E. Haloxylon ammodendron, F. Suaeda aralocaspica, G. S. eltonica, and H. S. maritima. Genes shown outside the outer circle are transcribed clockwise whereas those represented inside are transcribed counterclockwise. Large single copy (LSC), small single copy (SSC), and inverted repeats (IRa, IRb) regions are indicated.

## Slide 2
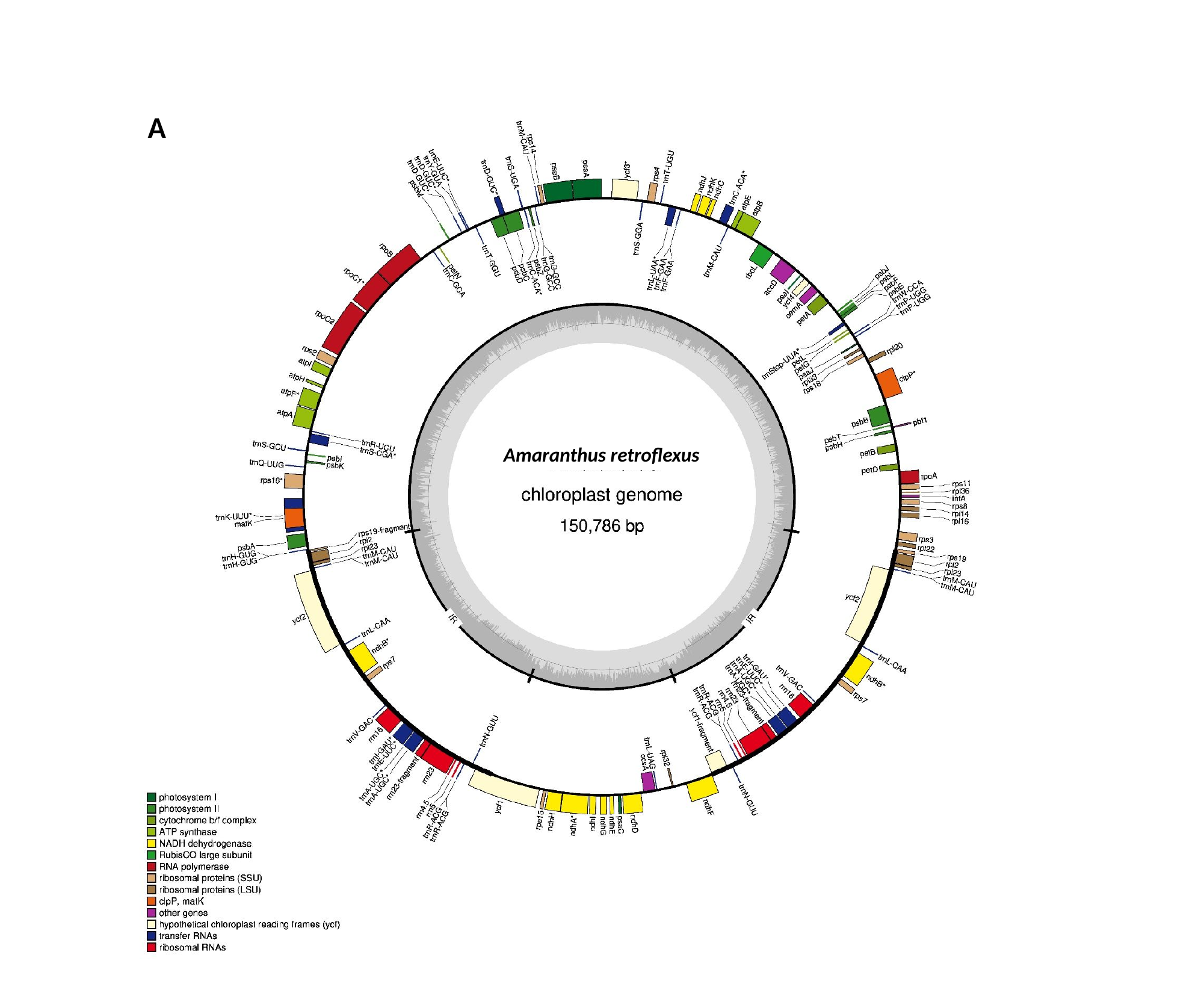

Amaranthus retroflexus
A

## Slide 3
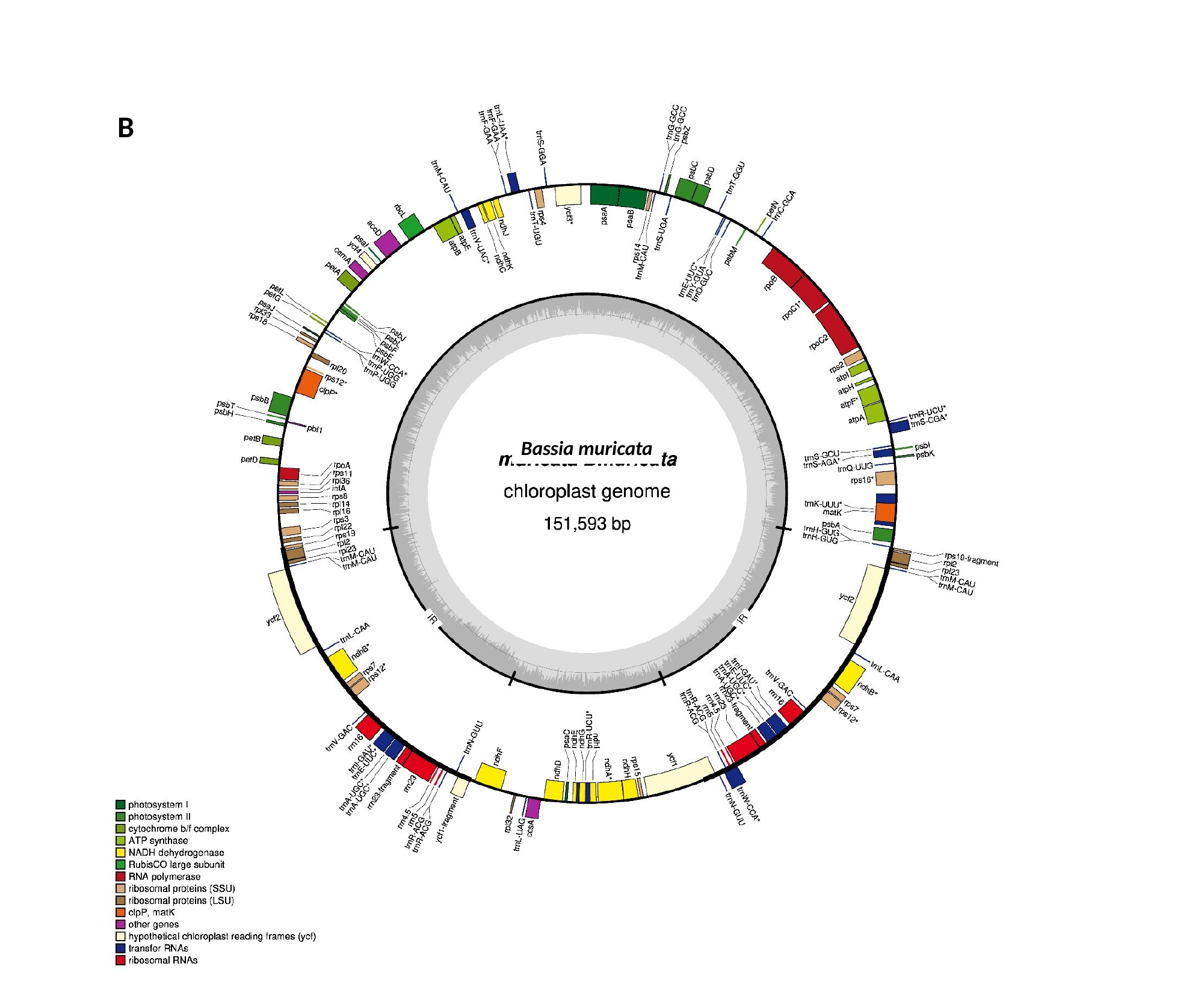

Bassia muricata
B

## Slide 4
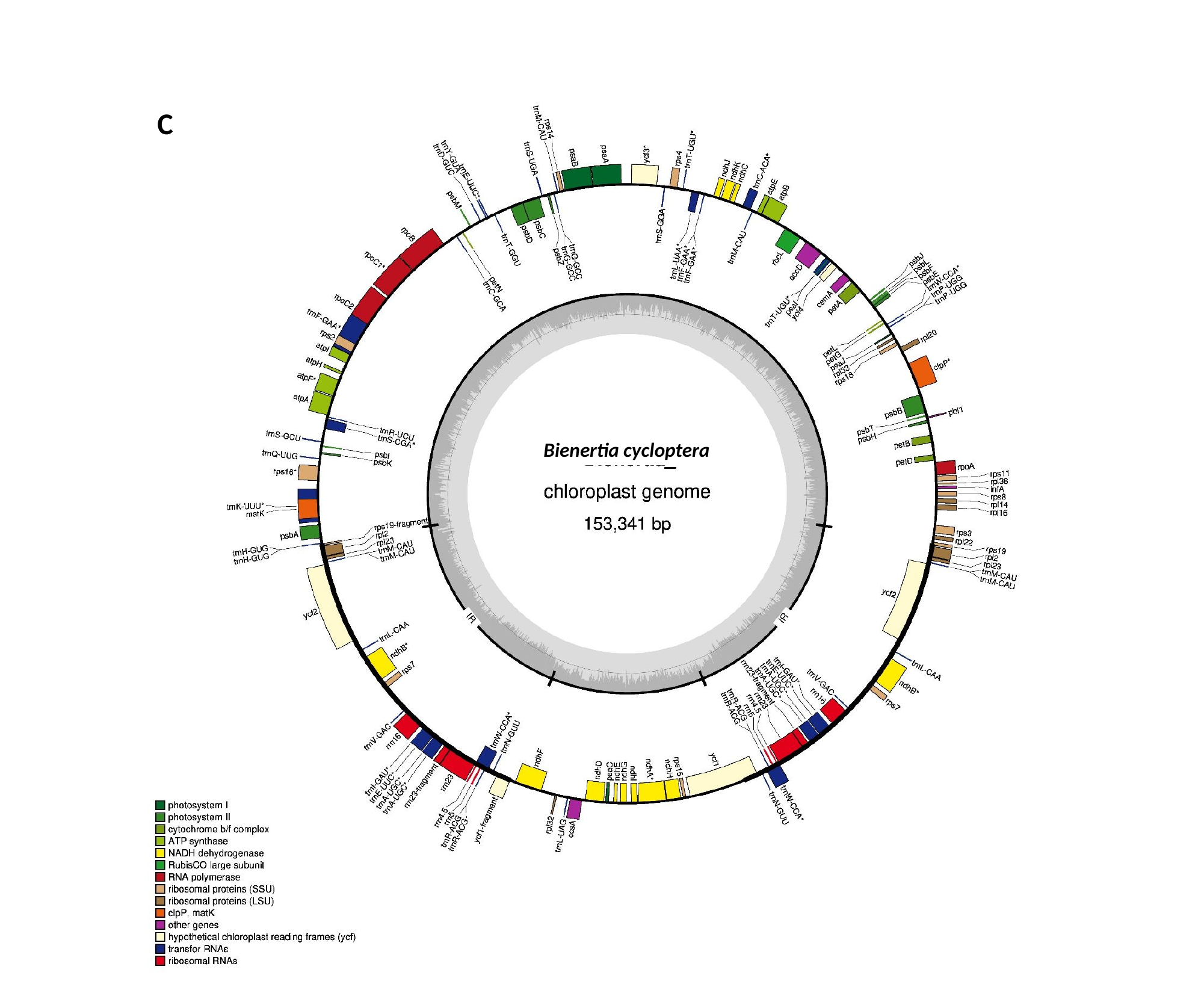

Bienertia cycloptera
C

## Slide 5
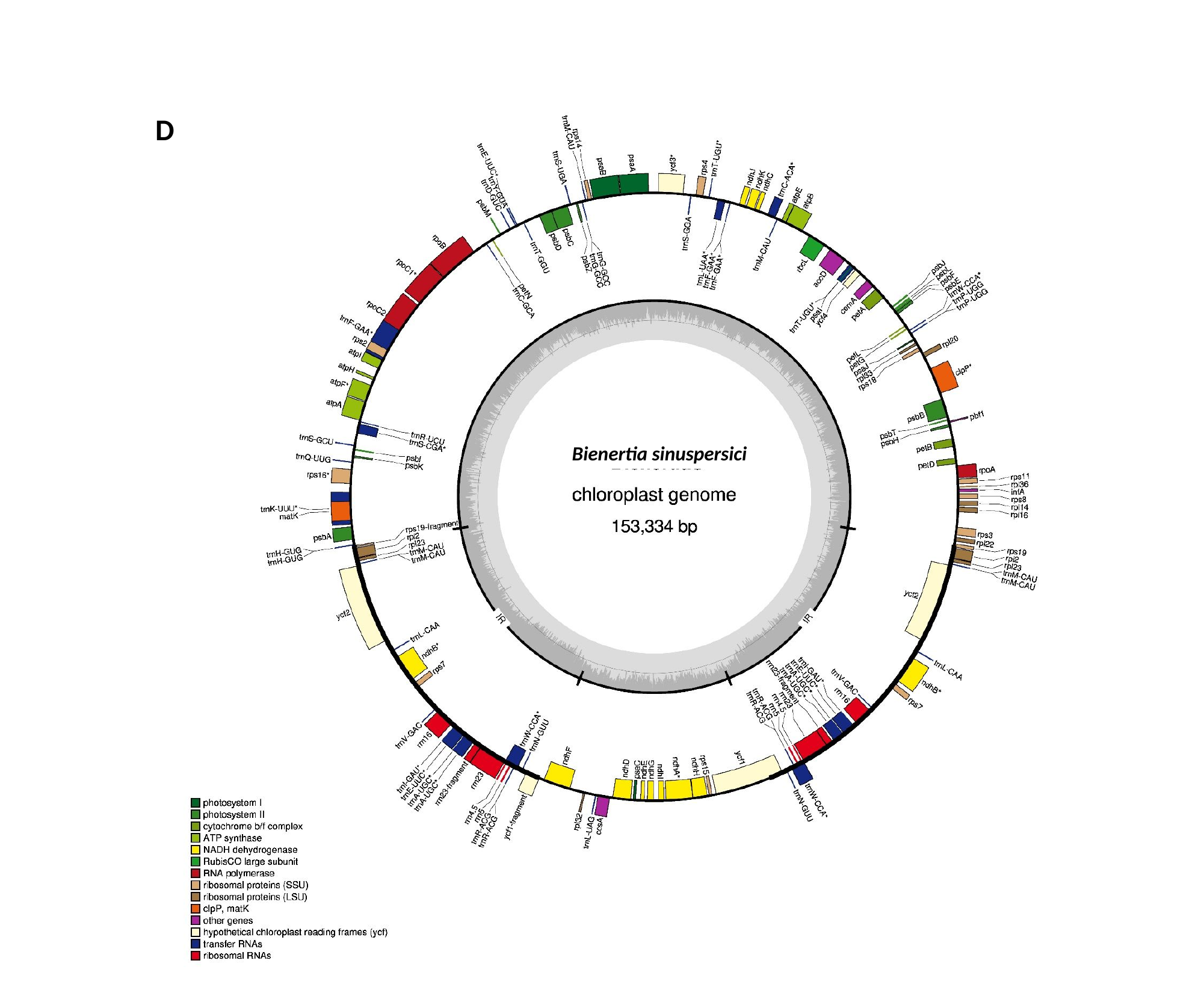

Bienertia sinuspersici
D

## Slide 6
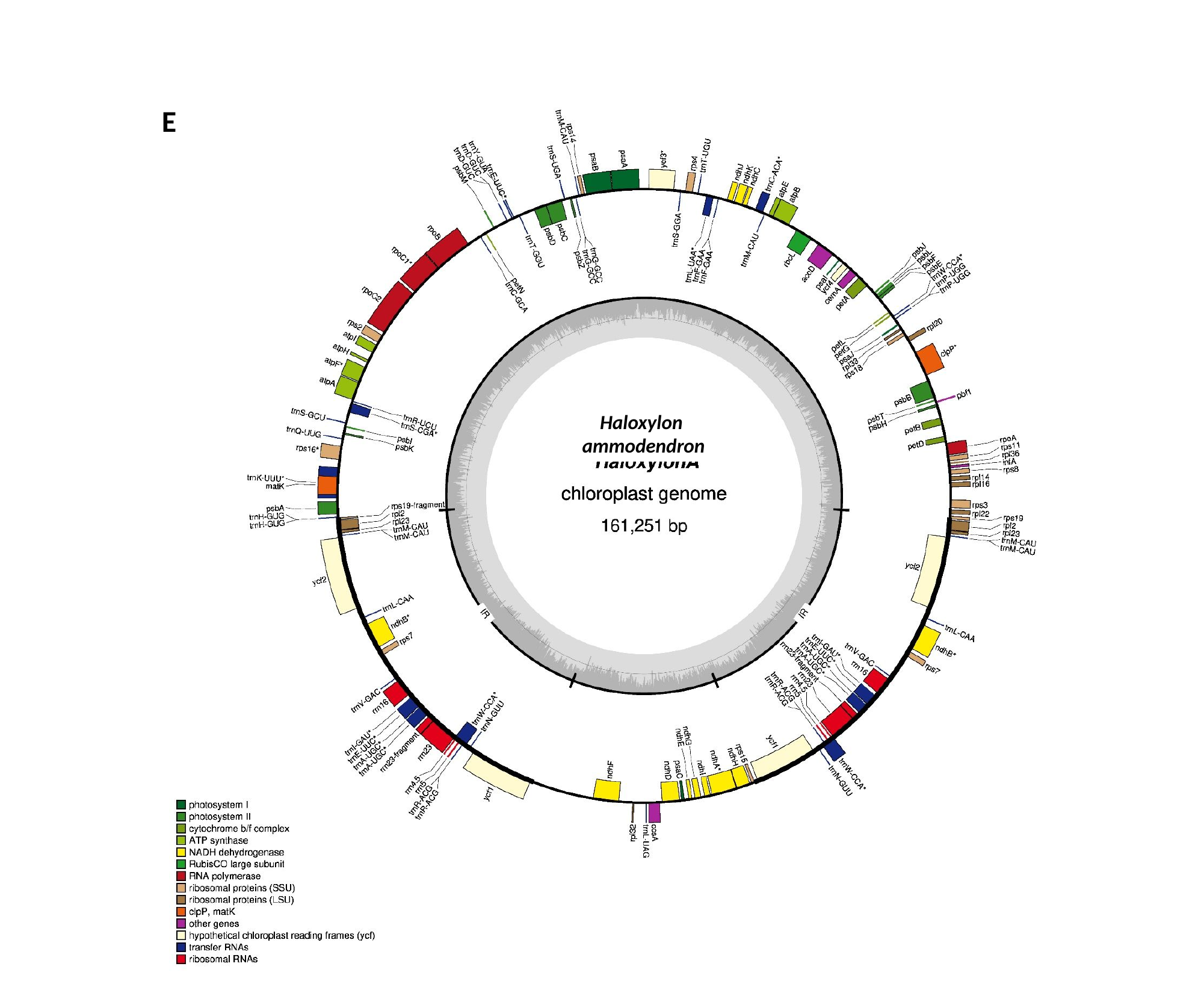

Haloxylon
ammodendron
E

## Slide 7
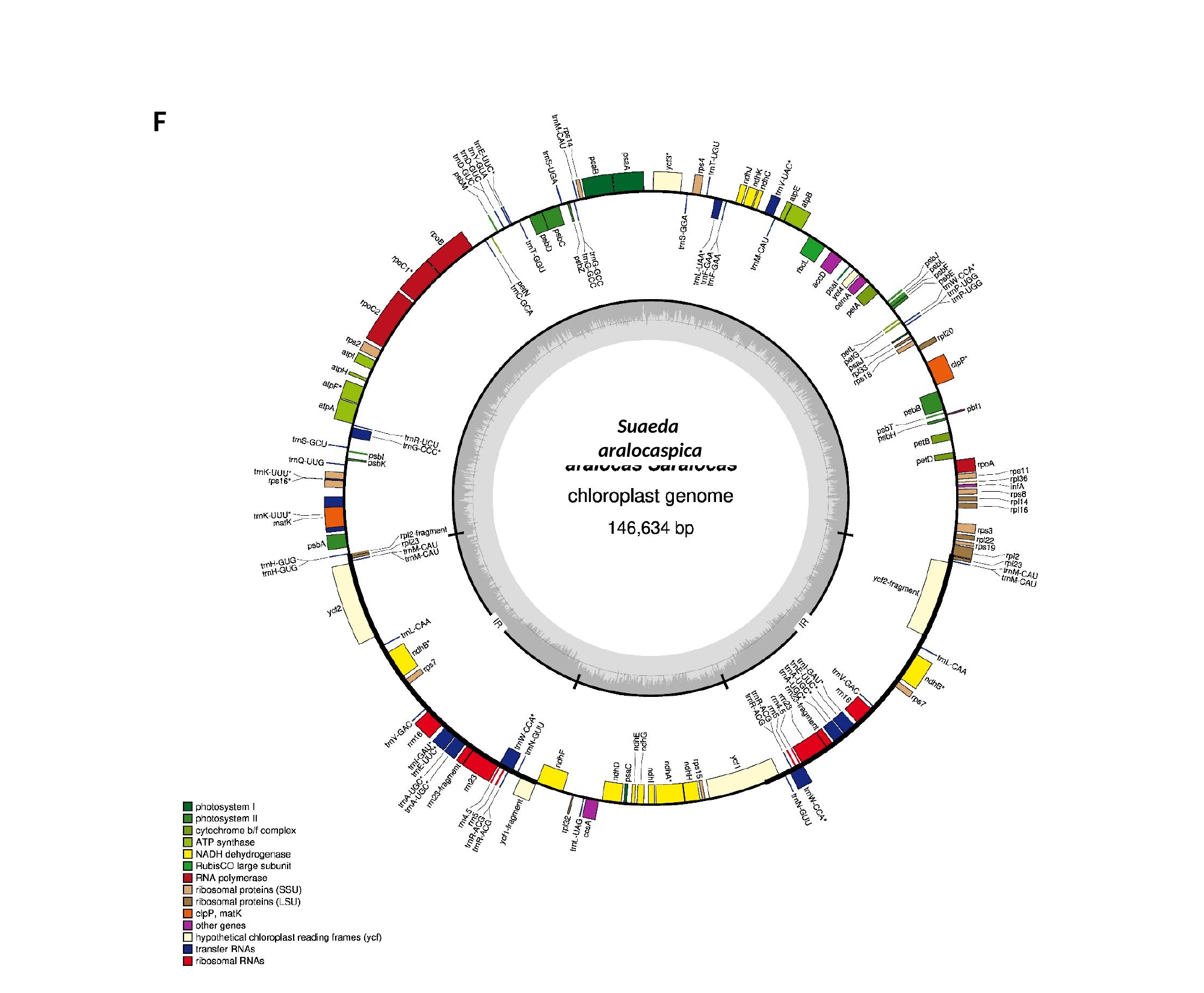

Suaeda
aralocaspica
F

## Slide 8
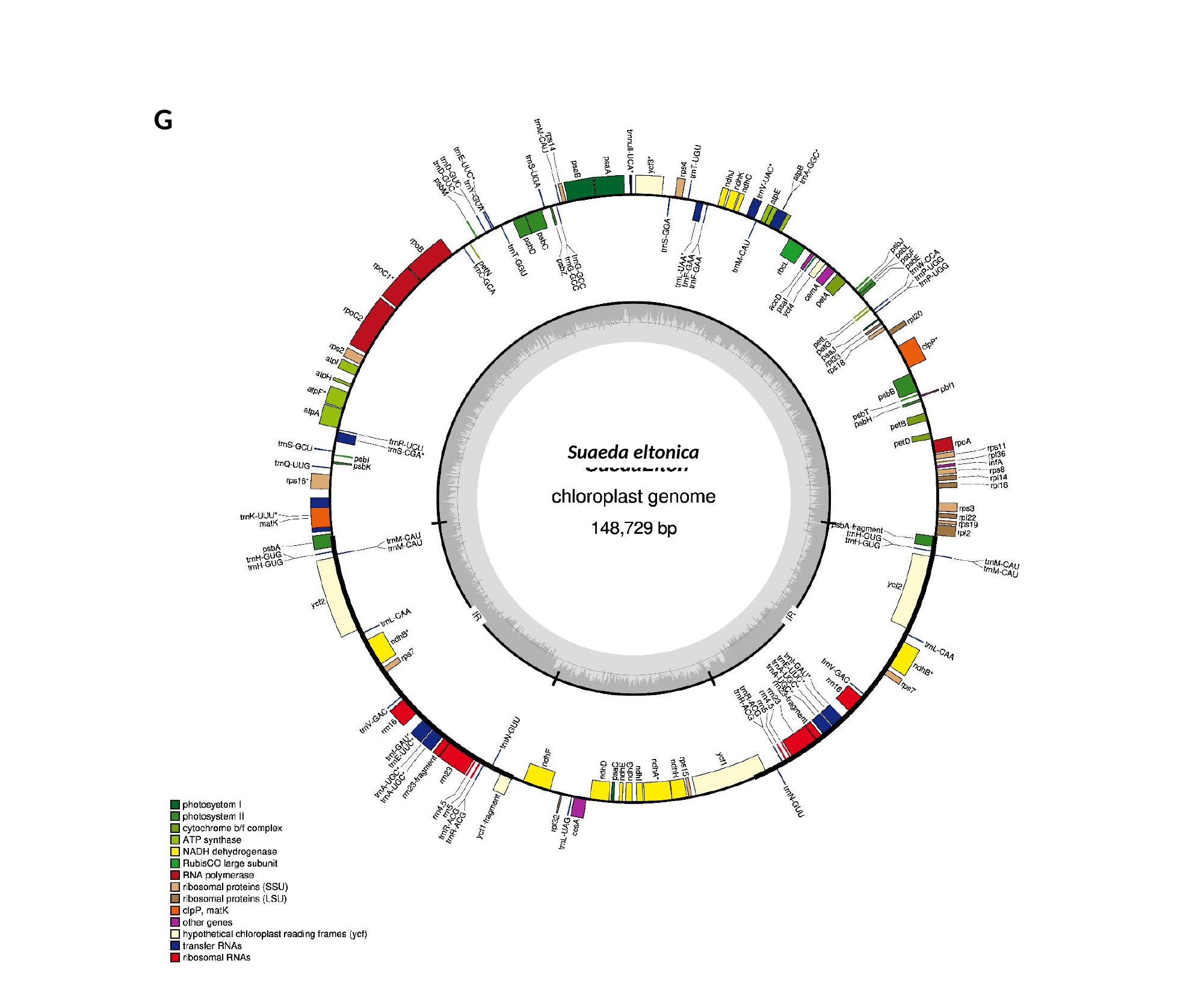

Suaeda eltonica
G

## Slide 9
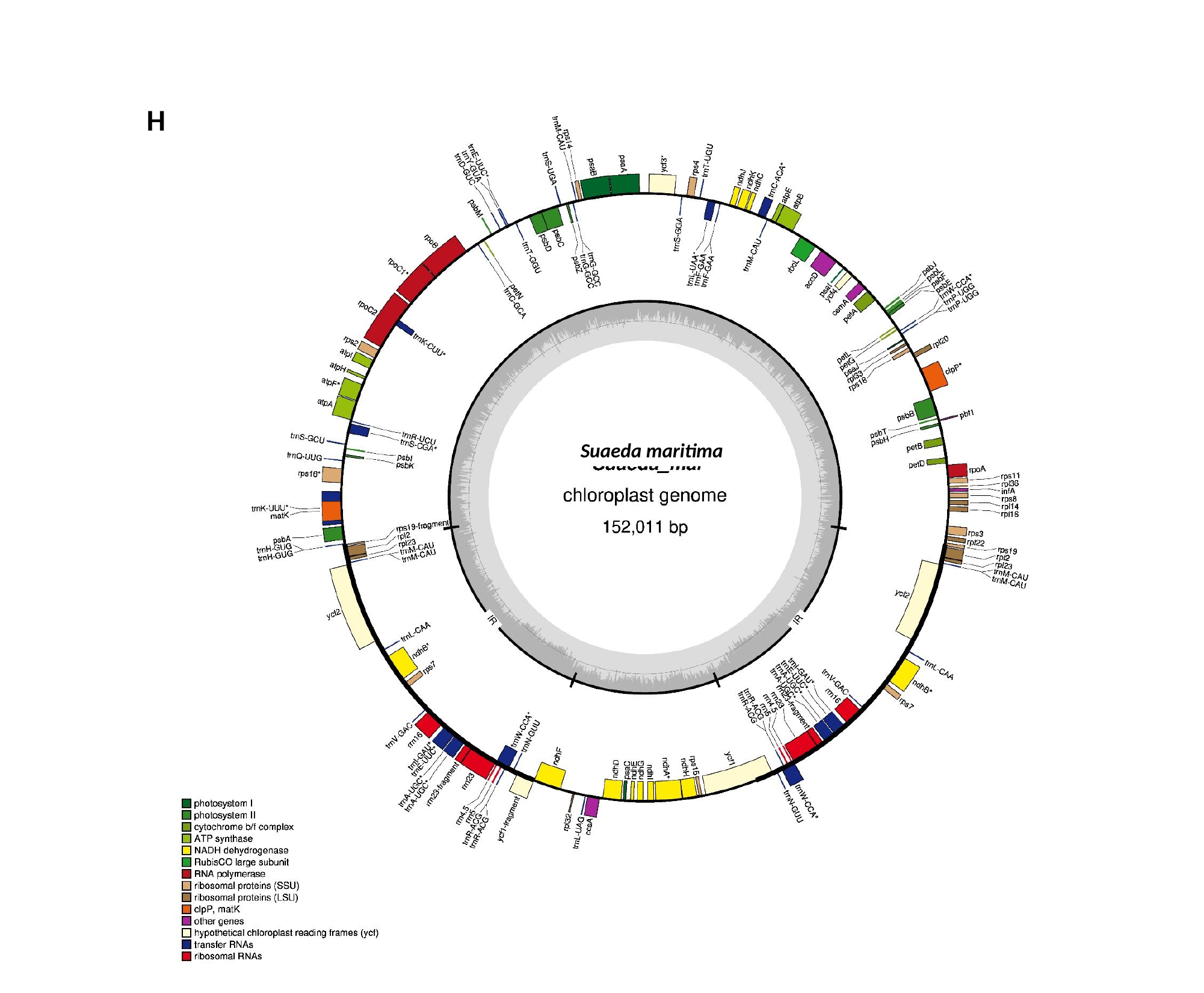

Suaeda maritima
H
