# Supplementary material for: Methods of analysis of chloroplast genomes of C3, Kranz type C4 and Single Cell C4 photosynthetic members of Chenopodiaceae
Source: Plant Methods. 2020 Aug 31;16:119. doi: 10.1186/s13007-020-00662-w (PMC7457496; doi:10.1186/s13007-020-00662-w)
Supplement: Supplementary file 5 — Additional file 5: Table S2. Forward and reverse primers used to amplify and validate the mitochondrion-to-plastidial DNA transfer in Haloxylon ammodendron and H. persicum. [file 13007_2020_662_MOESM5_ESM.pptx]

## Slide 1
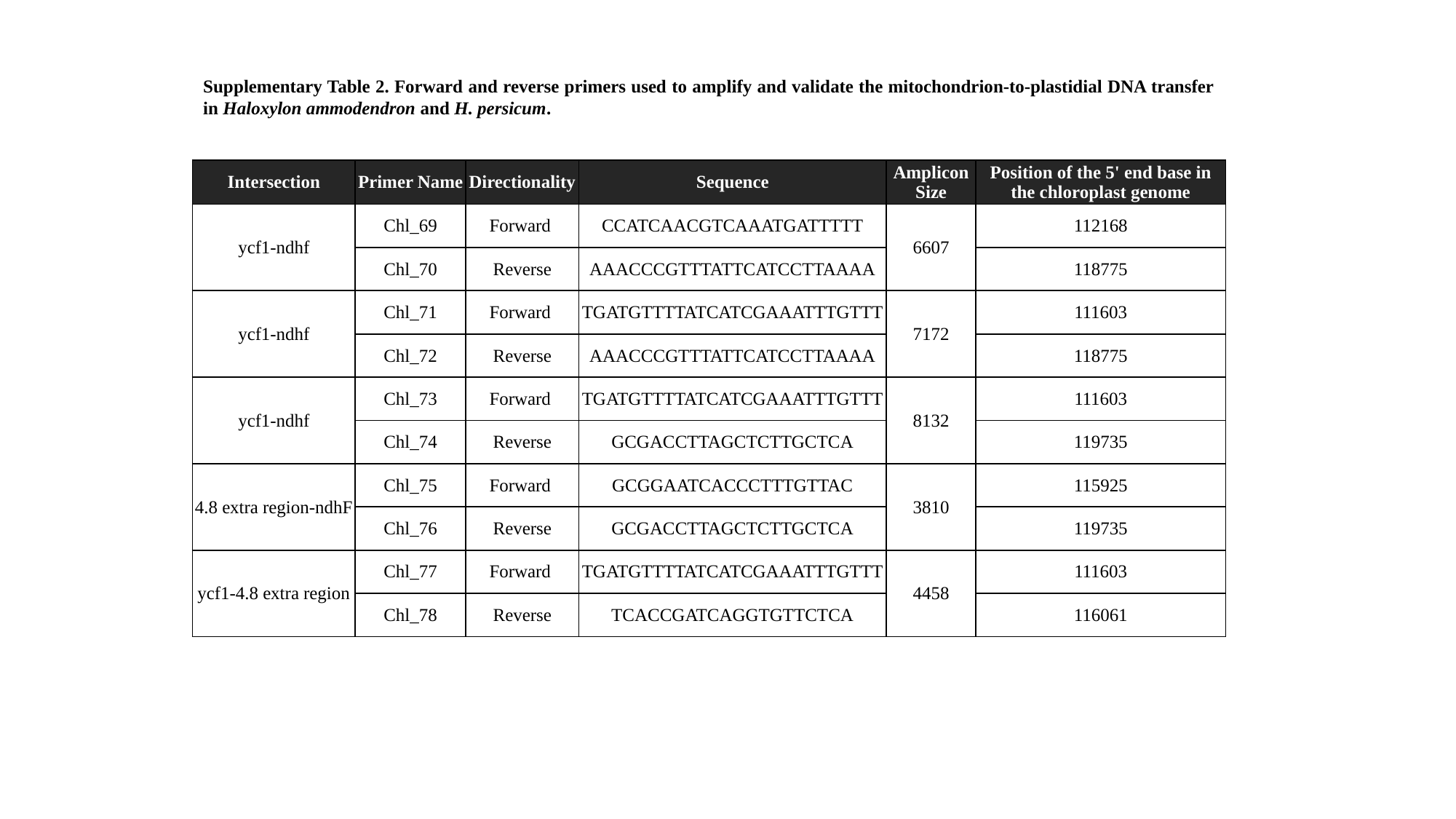

Supplementary Table 2. Forward and reverse primers used to amplify and validate the mitochondrion-to-plastidial DNA transfer in Haloxylon ammodendron and H. persicum.
| Intersection | Primer Name | Directionality | Sequence | Amplicon Size | Position of the 5' end base in the chloroplast genome |
| --- | --- | --- | --- | --- | --- |
| ycf1-ndhf | Chl\_69 | Forward | CCATCAACGTCAAATGATTTTT | 6607 | 112168 |
| | Chl\_70 | Reverse | AAACCCGTTTATTCATCCTTAAAA | | 118775 |
| ycf1-ndhf | Chl\_71 | Forward | TGATGTTTTATCATCGAAATTTGTTT | 7172 | 111603 |
| | Chl\_72 | Reverse | AAACCCGTTTATTCATCCTTAAAA | | 118775 |
| ycf1-ndhf | Chl\_73 | Forward | TGATGTTTTATCATCGAAATTTGTTT | 8132 | 111603 |
| | Chl\_74 | Reverse | GCGACCTTAGCTCTTGCTCA | | 119735 |
| 4.8 extra region-ndhF | Chl\_75 | Forward | GCGGAATCACCCTTTGTTAC | 3810 | 115925 |
| | Chl\_76 | Reverse | GCGACCTTAGCTCTTGCTCA | | 119735 |
| ycf1-4.8 extra region | Chl\_77 | Forward | TGATGTTTTATCATCGAAATTTGTTT | 4458 | 111603 |
| | Chl\_78 | Reverse | TCACCGATCAGGTGTTCTCA | | 116061 |
